# Progress in RNA‐Targeted Therapeutics for Human Diseases

**DOI:** 10.1002/mco2.70607

**Published:** 2026-02-05

**Authors:** Wangzheqi Zhang, Aimin Jiang, Bin‐Kui Jia, Yuming Jin, Yinghu Chen, Zhaoyu Li, Yan Liao, Haoling Zhang, Zhiheng Lin, Xiao Fang, Linhui Wang

**Affiliations:** ^1^ Department of Urology Changzheng Hospital, Naval Medical University (Second Military Medical University) Shanghai China; ^2^ Faculty of Anesthesiology Changhai Hospital, Naval Medical University (Second Military Medical University) Shanghai China; ^3^ Department of Urology No. 905 Hospital of PLA Navy, Naval Medical University (Second Military Medical University) Shanghai China; ^4^ Department of Urology Changhai Hospital, Naval Medical University (Second Military Medical University) Shanghai China; ^5^ Department of Urology The First Naval Hospital of Southern Theater Command Zhanjiang China; ^6^ Clinical College of Traditional Chinese Medicine Gansu University of Chinese Medicine, Gansu University of Chinese Medicine Lanzhou Gansu Province China; ^7^ College of Acupuncture‐Moxibustion and Tuina Gansu University of Chinese Medicine Lanzhou Gansu Province China; ^8^ Department of Biomedical Sciences Advanced Medical and Dental Institute, Universiti Sains Malaysia Penang Malaysia; ^9^ Department of Gynecology Longhua Hospital, Shanghai University of Traditional Chinese Medicine Shanghai China

**Keywords:** antisense oligonucleotides, CRISPR/Cas9, messenger RNA, RNA‐targeted therapy, small interfering RNA

## Abstract

RNA‐targeted therapy is reshaping molecular medicine by shifting the traditional “protein‐centric” view toward an “RNA‐regulatory network” paradigm. Beyond carrying genetic information, RNA plays essential roles in posttranscriptional regulation, signaling pathways, and epigenetic modulation. Advances in high‐throughput sequencing, structural biology, and delivery technologies have accelerated the development of diverse RNA therapeutics, including antisense oligonucleotides (ASOs), small interfering RNA (siRNA), microRNA (miRNA) modulators, messenger RNA (mRNA) therapeutics, aptamers, short hairpin RNA, and CRISPR/Cas‐guided single‐guide RNAs. However, a concise comparison of these major RNA modalities and the translational barriers that limit their broader clinical application is still lacking. This review outlines the mechanisms and representative applications of these RNA‐based strategies in gene silencing, editing, protein replacement, immune activation, and targeted drug delivery. Special emphasis is placed on ASOs and siRNAs for neurological, metabolic, and infectious diseases, as well as mRNA therapeutics that are transforming vaccine development. Common challenges‐such as in vivo stability, delivery efficiency, and immune activation‐are also discussed. Finally, we highlight how chemical modification, nanotechnology, and artificial intelligence‐assisted design are enhancing the specificity, stability, and safety of RNA therapeutics, providing a framework for advancing next‐generation precision RNA medicine.

## Graphical abstract

RNA‐targeted therapies are revolutionizing molecular medicine by transitioning from a “protein‐centric” focus to an “RNA‐regulatory network” approach. Leveraging RNA's diverse roles in gene regulation, signaling, and epigenetic modifications, advanced platforms such as ASOs, siRNA, miRNA, mRNA, aptamers, shRNA, and CRISPR/Cas systems are enabling precise gene silencing, editing, protein replacement, and immune activation. These strategies show promise in treating neurodegenerative, metabolic, infectious, and rare diseases, as well as cancers and inflammatory conditions. Despite challenges in stability, delivery, and safety, innovations in chemical modifications, nanotechnology, and AI‐driven design continue to optimize RNA stability, specificity, and immunogenicity. RNA therapeutics offer groundbreaking potential for precision molecular interventions in complex diseases.

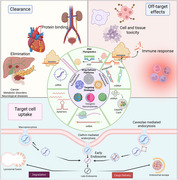

## 1 Introduction

RNA has emerged as a central regulator of gene expression, cellular homeostasis, and disease pathogenesis, extending well beyond its classical role as a messenger between DNA and proteins. Advances in transcriptomics have revealed a complex RNA regulatory network comprising both coding and noncoding transcripts, which together influence signal transduction, epigenetics, and posttranscriptional regulation (PTR). PTR maintains gene expression homeostasis by modulating the stability, abundance, and sequence of mature RNA [1]. Dysregulation of RNA expression and function is closely associated with cancer [2, 3], neurodegenerative diseases [4, 5], metabolic disorders, and immune dysregulation [6], highlighting the potential of RNA molecules as promising therapeutic targets. For example, in lung adenocarcinoma, key genes such as MCF2, identified through their role in hypoxia‐induced anoikis resistance, represent promising targets for RNA‐based therapies like antisense oligonucleotides (ASOs) or small interfering RNAs (siRNAs) [7]. In contrast to traditional protein‐targeted drugs, RNA‐targeted therapies directly modulate pathogenic RNAs at the gene expression level. Through sequence‐specific recognition, these therapies enable precise silencing, splicing regulation, or protein restoration, circumventing the challenges of drug development related to protein structure and “undruggable” targets. This “RNA network‐level regulation” offers superior designability, scalability, and target coverage compared with conventional small molecules and monoclonal antibodies.

Over the past two decades, the concept of RNA‐targeted therapies has evolved rapidly. Early breakthroughs, such as ASOs and RNA interference (RNAi), laid the foundation for sequence‐specific gene silencing. The clinical success of ASO and siRNA‐based drugs has validated the feasibility of achieving therapeutic benefits through RNA modulation. More recently, driven by the robust success of messenger RNA (mRNA) vaccines, new RNA‐based therapies, including mRNA technologies, RNA aptamers, microRNA (miRNA) modulators, and CRISPR/Cas‐guided single‐guide RNAs (sgRNAs), have been scaled up, significantly expanding the therapeutic landscape [8, 9]. Concurrently, innovations in chemical modifications, lipid nanoparticles (LNPs), N‐acetylgalactosamine (GalNAc) conjugation, and exosome‐based delivery technologies have further improved the stability, safety, and tissue specificity of RNA drugs. The global RNA drug development pipeline has seen a notable acceleration in recent years. Several siRNA and ASO drugs have been approved for disease treatment, mRNA technology has expanded its applications in vaccines and protein replacement therapies [10], and CRISPR/Cas‐based in vivo gene editing therapies are entering clinical validation. Additionally, the rapid advancement of tissue‐specific delivery technologies such as GalNAc conjugation, LNPs, polymeric nanoparticles, and engineered exosomes has facilitated the extension of RNA drugs from rare diseases to more common multisystem disorders. Notably, the development of aminourea–LNPs highlights their enormous potential for organ‐targeted mRNA delivery and cancer immunotherapy, opening new directions for RNA drug applications [11]. These trends indicate that RNA‐targeted therapies are transitioning from single‐target interventions to a systemic precision medicine platform.

Despite these advancements, the field still lacks a comprehensive comparative study that integrates the major RNA therapeutic strategies—examining their mechanisms of action, representative disease applications, and the common translational challenges hindering their broader clinical implementation. Existing reports often focus on individual RNA therapies or specific diseases, leaving a gap in understanding the shared principles, advantages, and limitations across platforms.

**FIGURE 1:**
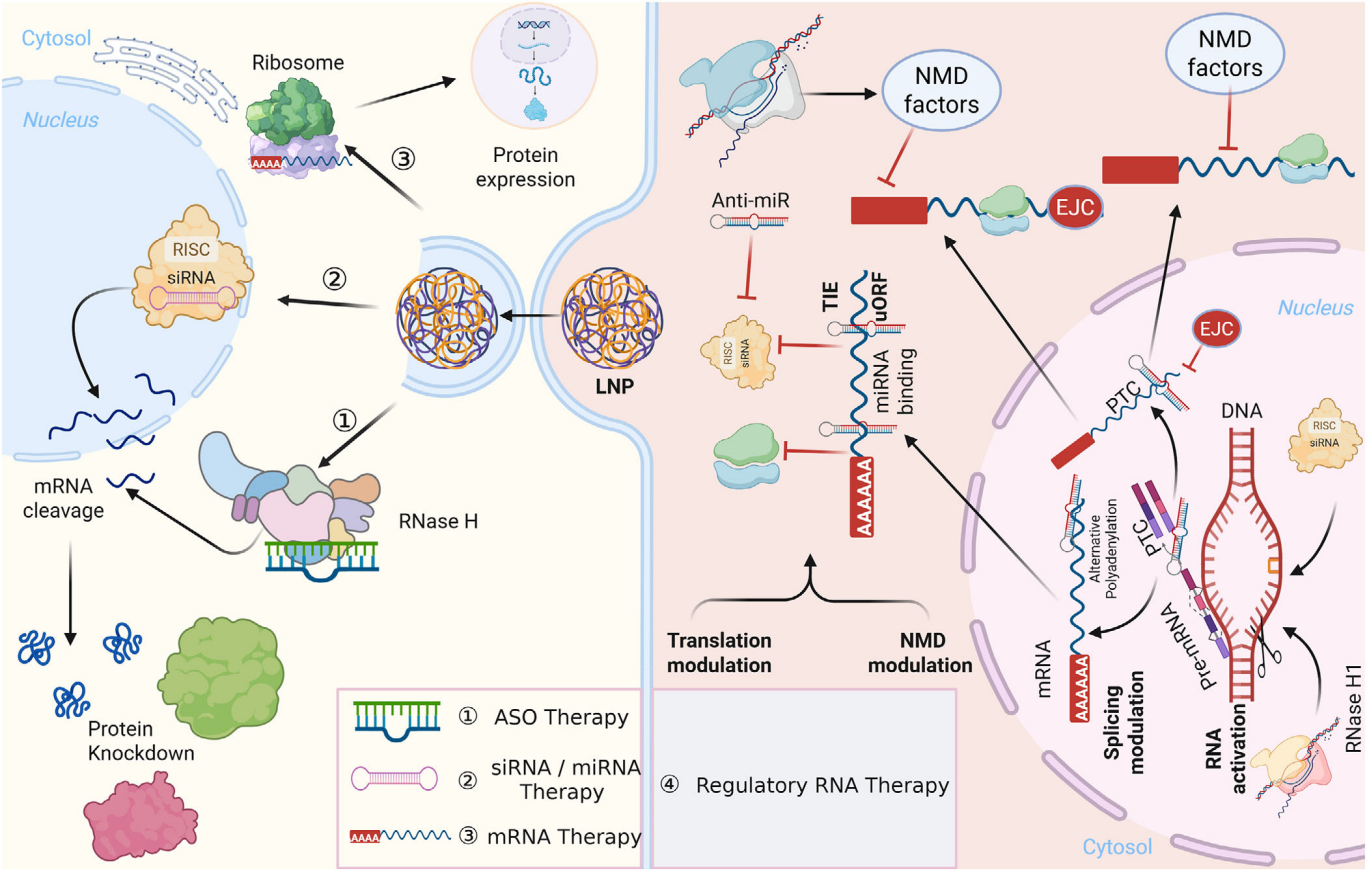
Historical development and core molecular mechanisms of RNA‐targeted therapeutics. This figure outlines the mechanistic basis underlying major classes of RNA‐targeted therapies, including ASOs, siRNAs/miRNAs, and mRNA‐based therapeutics. ASOs induce RNase‐H‐mediated degradation of complementary mRNA transcripts, whereas siRNAs and miRNAs guide the RISC complex to silence target genes through sequence‐specific mRNA cleavage. mRNA therapeutics, typically delivered via LNPs, are translated into functional proteins after cytoplasmic entry. Together, these modalities illustrate the progression from gene silencing to protein restoration, highlighting the diverse posttranscriptional mechanisms leveraged for therapeutic intervention. ASO, antisense oligonucleotide; siRNA, small interfering RNA; miRNA, microRNA; mRNA, messenger RNA; RISC, RNA‐induced silencing complex; RNase H, ribonuclease H; LNP, lipid nanoparticle.

This review aims to bridge this gap by providing a thorough and comparative overview of current RNA‐targeted therapies. We summarize the pharmacological mechanisms and therapeutic logic of RNAi platforms based on ASOs and siRNAs, examine the expanding applications of RNA aptamers and mRNA therapies, analyze miRNA‐guided and short hairpin RNA (shRNA)/sgRNA‐mediated interventions, and highlight their representative applications in neurological, metabolic, infectious, and oncological diseases. Figure 1 illustrates the timeline and technological milestones of RNA drugs, from discovery and optimization to clinical translation, in chronological order. Finally, we consolidate the common challenges faced, including delivery, specificity, immunogenicity, and long‐term safety, and discuss how chemical engineering, nanotechnology, and artificial intelligence (AI)‐assisted design will drive the development of next‐generation precision RNA drugs.

## 2 ASO‐Based Targeted Therapy

ASOs represent one of the most clinically advanced RNA‐targeted platforms and provide a paradigm for gene‐specific modulation at the RNA level. In this section, we first summarize the chemical features and mechanisms of action of different ASO subclasses, then highlight representative disease‐targeted applications across neurological, metabolic, inflammatory and infectious disorders, and finally discuss key opportunities and challenges related to target specificity, cellular uptake, and delivery strategies.

### 2.1 Introduction and Mechanisms of Action

ASOs are short nucleic acid sequences that hybridize to complementary RNA using base‐pairing interactions similar to Watson–Crick interactions. ASOs are typically made up of small chains of 18–30 nucleotides [12, 13]. These oligonucleotides they can accurately recognize and bind to target mRNA or precursor mRNA (pre‐mRNA). So the gene expression is regulated by ASOs via inhibiting, resplicing or degrading the target RNA. Therapeutic modalities that target RNA include ASOs, which are one of the most established and clinically validated approaches. Unlike small molecule drugs or monoclonal antibodies that mainly act at the protein level, ASOs work at the nucleic acid level, allowing for high sequence specificity and structural programmability. This thereby potentially allow for the fine‐tuned regulation of gene expression, potentially providing effective treatments for genetic, metabolic, and neurological diseases [13].

The biological activity of ASOs, as well as the downstream pathway that are activated upon hybridization to target RNA, is significantly influenced by the chemical structure of the ASOs [14]. RNase H‐dependent ASOs are a major class of ASOs that generate a DNA–RNA heteroduplex with the complementary mRNA target. The binding of the DNA strand by RNase H results in the degradation of the RNA strand and an efficient inhibition of the harmful protein production [14]. One such example is nusinersen, the first United States Food and Drug Administration‐approved ASO therapy for SMA, which has this mechanism of action. Another subclass are the splicing‐modulatory ASOs, which bind to splice sites or other regulatory elements in the pre‐mRNA to alter patterns of inclusion and exclusion of exons to restore or suppress the expression of a protein. For instance, eteplirsen encourages the skipping of exons in the gene associated with DMD, allowing the regional re‐establishment of functional dystrophin protein expression in DMD patients. Third, posttranscriptional inhibitory ASOs control gene expression by blocking mRNA cap formation, interfering with the assembly of translation initiation complexes, or competing for miRNA binding sites, thereby providing a versatile means for the fine‐tuning of translational output. Furthermore, some ASOs can travel to the nucleus, where they interact with lncRNAs or circRNAs that regulate their transcriptional or regulatory activity. This is an extension of this RNA therapeutic platform beyond just mRNA.

To improve the stability, bioavailability, and pharmacokinetic properties of ASOs in vivo, several chemical modification strategies are adopted. Examples of modifications: phosphorothioate backbone substitution, 2′‐MOE and LNA modifications are commonly used, which improve nuclease resistance, binding affinity and plasma half‐life [15]. Developments in delivery systems such as LNPs, GalNAc conjugation, and polyethylene glycol (PEG) modification allow the modulation of tissue specificity, particularly to the central nervous system (CNS) or liver. The use of ASOs is also highly beneficial due to their ease of design, efficient synthesis, and prolonged in vivo activity, which permits a quick response to new pathogenic targets or mutations. These qualities offer outstanding adaptability as well as translational potential for drug development [16]. ASO technology has implications for human disease. Antimicrobial applications targeting small bacterial RNA like MicF, which adjusts the expression of outer membrane protein OmpF, show promise for development using ASO technology.

**FIGURE 2:**
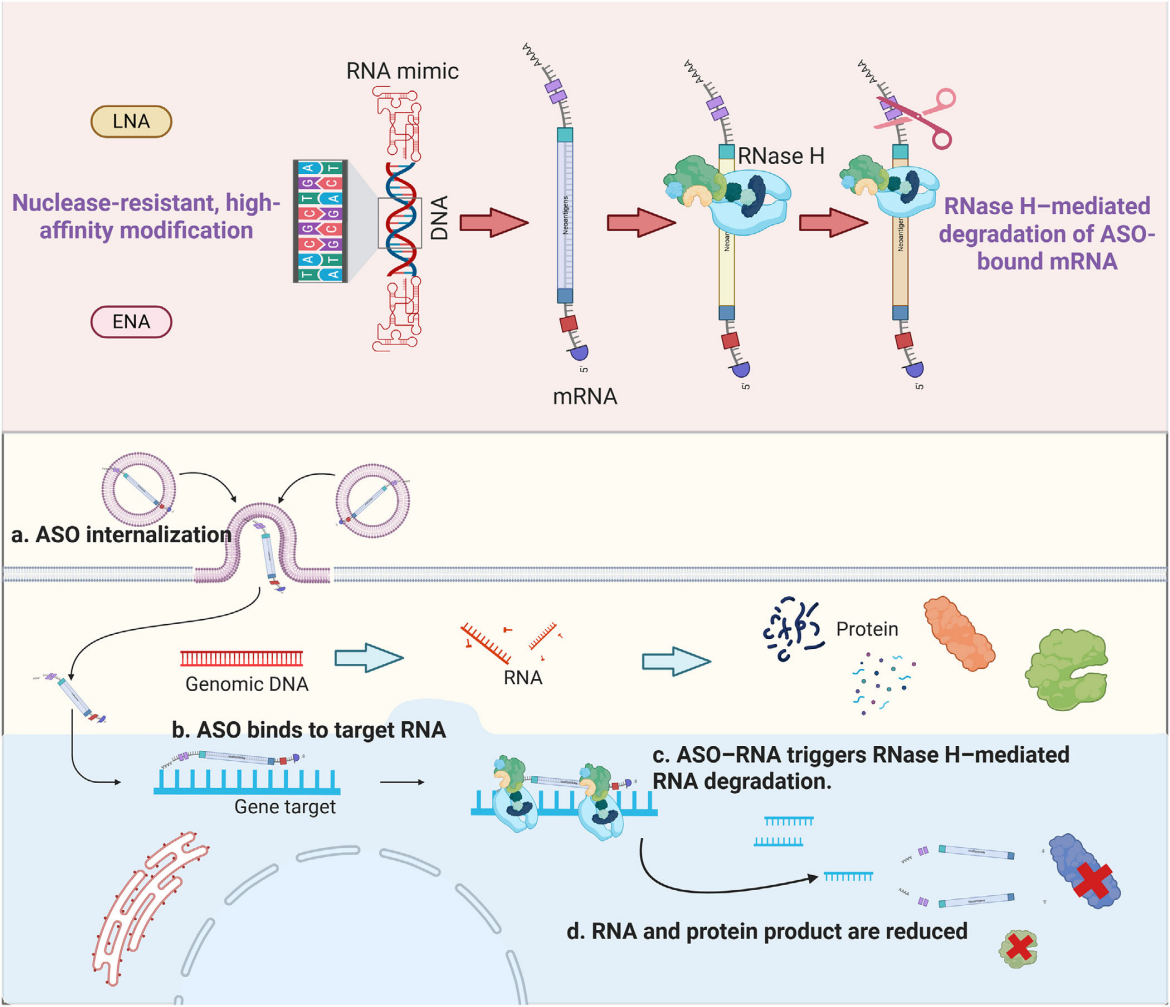
Mechanistic actions of chemically modified ASOs in RNase‐H‐mediated gene silencing. The image shows how chemical modifications such as LNA and ENA enhance the nuclease resistance and hybridization affinity of ASOs toward target mRNA. Upon entry into the cell, ASOs bind to the targeted RNA via complementary sequences. This binding allows the formation of DNA–RNA heteroduplexes that recruit RNase H to cleave the mRNA transcript selectively. The result indicates that RNA quantity and subsequent protein production are decreased by the process, thus ASO therapeutics have sequence‐specific, posttranscriptional regulatory capacity. ASO, antisense oligonucleotide; LNA, locked nucleic acid; ENA, ethylene‐bridged nucleic acid; mRNA, messenger RNA; RNase H, ribonuclease H.

This modulation alters antibiotic permeability and can reverse antimicrobial resistance or inhibit pathogen growth [17, 18]. The continuous optimization of ASO design and delivery has established this technology as a cornerstone of the emerging field of nucleic acid‐based therapeutics, providing innovative molecular intervention strategies for rare, infectious, and treatment‐refractory diseases. By specifically binding to target mRNA, ASOs trigger RNase H‐mediated degradation or regulate alternative splicing, resulting in gene silencing or translational modulation. Figure 2 illustrates the key molecular mechanisms of ASO action and their functional pathways in RNA‐targeted intervention.

### 2.2 Disease‐Targeted Applications

ASOs, an RNA‐level gene regulation platform, exert their therapeutic actions via sequence‐specific hybridization with target mRNA or pre‐mRNA, thus causing silencing, skipping, or restoration of expression. Together, these mechanisms correct disease phenotypes from defective gene expression. Unlike traditional small‐molecule drugs or antibody therapies, ASOs have unique specificity and significant design flexibility, and they act directly at the nucleic acid level. Thus, they offer great hope in the treatment of genetic, neurological, metabolic, and infectious diseases. Therapeutics based on ASOs are continuously progressing due to constant innovations in chemical modifications and delivery systems and are entering clinical practice as a core component of RNA‐targeted therapy.

With respect to all therapeutic areas, ASOs are most advanced in their development and application for neurological disease. According to the study, involvement of the FUS (fused in sarcoma) gene mutations is one of the causative gene mutations of aggressive ALS. ASOs that target FUS can knock down expression of the mutant allele [19]. An example includes ION363, a FUS‐targeting ASO with nonallele‐specific silencing properties that effectively lowers FUS transcript and protein levels in the CNS, delaying motor neuron degeneration and disease pathology [20]. Within the context of SMA, intrathecal ASO delivery has been shown to be a safe and efficacious approach for early treatment [21]. The combined application of ASO‐P1 and ASO‐NUS has significantly increased the levels of full‐length SMN2 transcripts and enhanced SMN protein expression, which has in turn alleviated the pathological and functional deficiencies seen in SMA animal models [22]. Furthermore, ASOs specific to α‐synuclein (aSyn) can prevent the transcription of the SNCA gene, leading to decreased pathological aSyn aggregation and reversal of PD‐related neurodegenerative changes. This strategy presents a novel molecular intervention for neurodegenerative diseases [23].

ASO therapeutics have shown diverse and promising applications in metabolic, inflammatory, and infectious diseases. The modulation of autoregulatory pathways of Regnase‐1 increases the stability and inhibits the release of proinflammatory cytokines, which can be exploited as a fine‐tuning tool in inflammatory diseases [24]. In liver‐related metabolic disorders, ASOs modulate translational termination to enhance the effectiveness of read‐through small molecules for treatment of genetic diseases with nonsense mutations and improve outcomes in metabolic dysfunction [25]. Furthermore, impressive antitumor effects of the Mal2–G4–ASO have been recorded in a series of in vitro and in vivo experiments [26]. Moreover, it was observed that ASOs targeting ACE2 effectively prevent SAR‐CoV‐2 from invading host cells. Therefore, this could be a new strategy for prevention and treatment of COVID‐19 and other ACE2‐dependent viral infections [27].

The potential of ASOs in anticancer therapy has increasingly gained attention. STAT3‐targeting ASOs can modulate the tumor immune microenvironment by enhancing T‐cell infiltration and reducing T‐cell exhaustion, thus synergizing with immune checkpoint inhibitors to exert antitumor effects. This highlights that the ASO platform has transcended traditional areas such as metabolism and inflammation, becoming an indispensable component of precision cancer immunotherapy [28]. Notably, the function and activity of key targets like STAT3 and immune checkpoint proteins are themselves finely tuned by posttranslational modifications (PTMs), suggesting that combining RNA‐targeted expression control with PTM‐targeted functional modulation could be a powerful layered therapeutic strategy [29]. Based on the RNase H‐mediated mRNA degradation mechanism, ASOs can effectively achieve multidimensional regulation of key oncogenes. By developing novel drugs that can be cleaved by RNase H and simultaneously release multiple ASOs, researchers have successfully inhibited the expression of HER2, Akt, and Hsp27 in breast cancer models, demonstrating robust antiproliferative effects. This lays a solid foundation for the development of multitarget ASO therapies aimed at complex oncogenic pathways [30].

To sum up, ASOs have become a vital component of many RNA‐targeted therapies because of their remarkable specificity, versatility, and broad utility. As preclinical and clinical studies diversify into areas such as neurodegenerative disorders, metabolic complications, inflammation, and infectious diseases, ASO‐based approaches are heading toward personalized precision medicine. These advancements will allow for diverse molecular interventions and create more translational opportunities for various human diseases.

### 2.3 Opportunities and Challenges: Target Specificity and Cellular Uptake Efficiency

ASOs have been revolutionizing how physicians treat certain genetic and neurological diseases. Their sequence design can be programmed to target specific disease‐causing RNA mutations with high precision. Nonetheless, application in the clinic is challenged primarily by two problems: target specificity and cellular uptake. It is important to optimize these attributes in ASO design. They significantly impact the therapeutic efficacy and safety of ASO‐based drugs. These challenges also pose a major technical hurdle for RNA drug development.

When we talk about specific targets, ASOs modify posttranscriptional gene expression by binding to specific RNAs through Watson‐Crick base pairing. Some sequences of ASO may not completely match RNA segments that are not the intended targets. This could cause some unintended consequences [31, 32]. The type of off‐target interactions can result in disruption of gene expression, cytotoxicity or immune response, and drug safety and selectivity. Studies have shown that moderately lengthening ASO sequences can help reduce off‐target effects [32]. Chemical modifications, that is, 2′‐MOE, LNA, and phosphorothioate modifications have been found to enhance base‐pairing affinity and thermal stability [33]. In vitro, ASOs tend to generate nonspecific binding when present at high concentrations. In vivo, the gradual internalization and low concentrations contribute to high specificity [33]. Genomic sequence differences between humans and experimental animals do not allow the conventional use of animal models for simulating ASO‐induced off‐target toxicity, thus limiting preclinical safety studies [34, 35]. Consequently, forthcoming investigations need to be focused on using high‐resolution in vivo omics and computational simulations for the development of more humanized off‐target prediction systems.

ASO molecules are generally negatively charged, which limits their ability to penetrate cell membranes and hinders their cellular uptake and delivery. After endocytosis, ASOs are frequently trapped in endosomes or lysosomes, which prevents them from reaching effective concentrations in the nucleus [36, 37]. Endosomal escape is a key rate‐limiting step that limits the intracellular activity of ASOs [38, 39]. To address this problem, OECs (oligonucleotide‐enhancing compounds) have been developed. OECs promote the release of ASOs from endosomes into the cytoplasm, greatly increasing their nuclear concentration and thereby enhancing their regulatory efficiency on target mRNAs [38]. Moreover, advanced delivery systems, such as LNP, GalNAc conjugates, and cell‐penetrating peptides (CPPs), have enhanced the tissue specificity and bioavailability of ASOs.

LNP delivery strategies offer significant advantages in protecting nucleic acid drugs, but their in vivo distribution presents notable limitations. Studies have shown that after systemic administration, the majority of LNPs accumulate in the liver and spleen, resulting in insufficient exposure in nonhepatic organs such as the heart, skeletal muscle, and kidneys. Furthermore, the blood–brain barrier (BBB) nearly completely blocks the entry of conventional LNPs, which makes ASO drugs targeting the CNS highly dependent on invasive delivery methods like intrathecal or intraventricular injections. This significantly increases the difficulty of clinical application and the burden on patients [40]. Additionally, ionizable lipids and PEG modifications in LNPs can trigger complement activation, infusion‐related reactions, or dose‐limiting hepatotoxicity. Issues such as particle size heterogeneity, inadequate endosomal escape, and the reproducibility challenges in large‐scale production further restrict clinical use. Therefore, LNPs are not a universal delivery solution for ASOs. Future efforts should focus on developing next‐generation engineered carriers with higher tissue specificity, lower immunogenicity, and improved delivery efficiency.

In addition to chemical modification strategies, cellular uptake and transmembrane transport capacity are also critical factors determining the tissue specificity and intervention efficiency of ASOs. In recent years, significant breakthroughs have been made in the targeting capability of LNP systems through the rational design of ionizable lipid structures. Research has shown that incorporating biodegradable β‐propionate ester linkers into ionizable lipids, along with specific hydrophobic chain branches and polar headgroups, can precisely modulate the ζ‐potential of LNPs, influencing their protein corona composition and ultimately enabling efficient and selective mRNA delivery to extra‐hepatic organs such as the lungs and spleen. For example, the lipid A3T2C7 (CP‐LC‐1495) achieved 97.1% selectivity and high protein expression in the lungs, highlighting the immense potential of lipid structure programming for tissue‐specific enrichment [41]. Furthermore, CPPs, membrane fusion peptides, and virus‐mimicking nanoparticles have demonstrated significant transmembrane delivery capabilities. These technologies enhance endosomal escape or enable direct cytoplasmic entry, thereby increasing the nuclear concentration of ASOs. By combining these novel delivery techniques, the specificity and functionality of ASOs can be greatly enhanced without altering their sequence.

The off‐target effects of RNA‐targeted therapies can lead to adverse reactions across multiple organs and systems, including hepatotoxicity, immune activation, and coagulation abnormalities. Therefore, a more refined risk control system needs to be established in clinical practice. Current clinical management of RNA therapies employs a multilayered cautious approach to identify and minimize potential nonspecific gene silencing (off‐target effects) and the unpredictable systemic toxicity associated with it at early stages [42]. Moreover, shortening dosing periods and adopting reversible intervention strategies help mitigate the cumulative risks of long‐term exposure while maintaining therapeutic efficacy. From a regulatory perspective, oligonucleotide‐based therapies, such as mRNA drugs, are subject to more stringent evaluation standards than conventional drugs. This includes a comprehensive assessment of immune toxicity triggered by ionizable lipids and the mRNA itself, liver and renal damage risks due to hepatic accumulation and neutrophil infiltration, and off‐target effects resulting from shifts in gene expression profiles, with particular attention to species‐specific prediction uncertainties. Additionally, novel delivery systems like LNPs raise regulatory concerns regarding their long‐term biosafety, the biodegradability of endosomal escape materials (such as the development of metabolizable trehalose lipids), and the potential for innate/adaptive immune responses triggered by the nanoparticles themselves (e.g., anti‐PEG antibodies or NLRP3 inflammasome activation) [43]. To address these challenges, establishing a comprehensive safety monitoring system, which includes advanced in vitro models and long‐term follow‐up, along with standardized nonclinical and clinical evaluation frameworks, is a critical step toward the safe clinical translation of RNA‐targeted therapies.

Looking ahead, the development of next‐generation ASO therapies will leverage AI‐assisted sequence design to predict stereochemical structures with unprecedented precision and minimize off‐target toxicity. Additionally, overcoming the “liver bottleneck” remains a priority. Beyond GalNAc, the integration of novel bioconjugation strategies, such as antibody–oligonucleotide conjugates (AOCs) and peptide–ASO chimeras, holds promise for achieving extra‐hepatic delivery to tissues such as the heart, muscles, and CNS. These advancements will drive the expansion of ASO therapies from rare genetic diseases to broader applications in chronic conditions.

## 3 siRNA‐Based Targeted Therapy

siRNAs are the core effectors of RNAi and have become a powerful tool for sequence‐specific gene silencing in vivo. This section first introduces the fundamental principles and molecular mechanisms of siRNA‐mediated RNAi, then reviews disease‐targeted applications in cancer, metabolic, cardiovascular, neurological, and viral diseases, and finally examines opportunities and challenges focusing on delivery systems, tissue targeting and off‐target effects.

### 3.1 Fundamental Principles and Mechanisms of Action

siRNA is a short dsRNA molecule with 20–25 nucleotides in length that induces degradation of target mRNA to inhibit the expression of specific genes, serving as a key player in the RNAi pathway [44]. Fire and Mello discovered the RNAi mechanism within the organism C. elegans. siRNAs achieve high specificity and efficiency by mediating the cleavage of target mRNA through perfect sequence complementarity, making them an important technology for precise RNA‐level therapies. Since this discovery, the RNAi mechanism has been shown to be essential in the regulation of gene expression, viral defense, immune balance and tumor suppression.

The RISC complex carries out the RNAi process, with the AGO protein family, especially AGO2, acting as its core catalytic component [45, 46]. This process begins when Dicer, an RNase III endonuclease, recognizes large dsRNA precursors and cleaves them into 21 nucleotide‐long siRNA duplexes. The RISC contains duplexes as well as cofactors like AGO2, TNRC6A, PABPC4, and PRKRA. Within this complex, AGO2 with a functionality similar to the endonucleases also has the characteristics of RNase H. AGO2 cleaves the complementary mRNA [47, 48, 49]. The RISC assembly process selects the guide strand of the siRNA duplex to be retained and assembled into active RISC while releases and degrades the passenger strand. The mature RISC, directed by an antisense (or guide) strand, pairs with a complementary target mRNA and induces AGO2‐mediated cleavage of the mRNA. This cleavage effectively reduces the expression of the relevant target gene by inhibiting translation.

The RNAi pathway relies on the formation and activation of RISC for its function. The RNAi efficiency relies on the correct processing of dsRNA by Dicer and on the stable assembly of RISC in the cytoplasm [50]. Research shows that stalled RISC complexes and their interactions with ribosomes—not just the accumulation of cleaved mRNAs—can potentially contribute to the recycling of siRNA and efficiency of silencing [51]. In addition, the cooperative function of Dicer‐2 and its partner protein R2D2 enhances the directionality and efficiency of siRNA loading into AGO2 and strengthens gene‐silencing activity [48]. RISC can recognize and destroy the viral RNAs and aberrantly expressed endogenous mRNAs via this mechanism, enabling PTR and rectification of pathologies [52].

**FIGURE 3:**
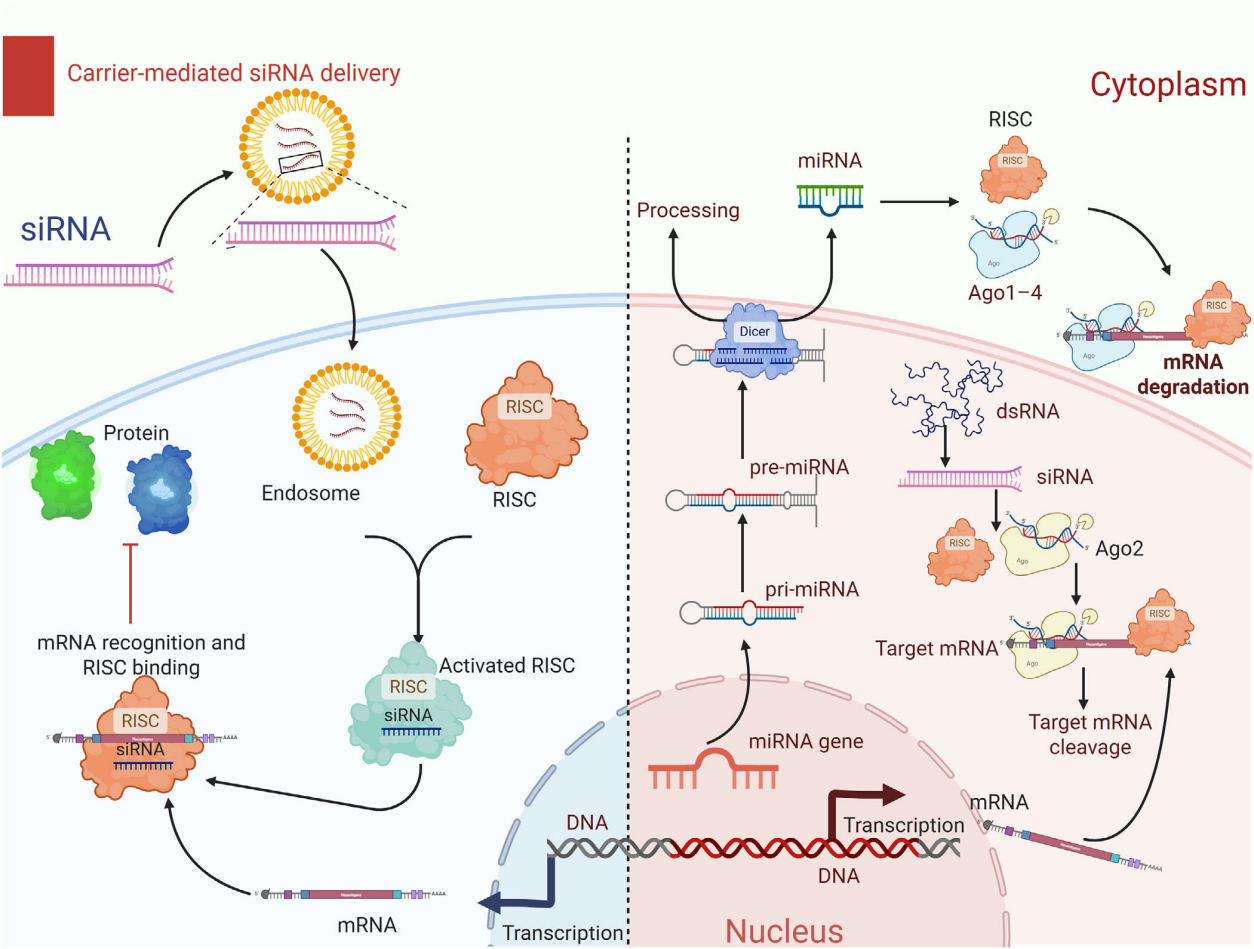
RNAi pathways mediated by synthetic siRNAs and endogenous miRNAs. The diagram depicts the process of carrier‐assisted delivery of synthetic siRNAs into the cytoplasm for loading into the RISC where they are incorporated into RISC to cleave a complementary target mRNA. Simultaneously, the endogenous pathway of miRNA is shown where nuclear transcription occurs and processing of pri‐miRNAs by Drosha takes place, followed by Dicer‐mediated maturation and incorporation into AGO‐containing RISC. The two pathways converge in a sequence‐guided posttranscriptional silencing via mRNA cleavage or translational repression. siRNA, small interfering RNA; miRNA, microRNA; RNAi, RNA interference; RISC, RNA‐induced silencing complex; mRNA, messenger RNA; pri‐miRNA, primary microRNA; AGO, argonaute; Drosha, RNase III endonuclease Drosha; Dicer, RNase III endonuclease Dicer.

siRNAs induce target mRNA degradation through a two‐step mechanism involving Dicer‐processing and RISC‐mediated cleavage of dsRNA precursors. The molecular basis of RNAi technology and RNA‐targeted therapeutics is the catalytic activity of AGO2 and the sequence specificity of the guide strand. Due to improved understanding of the structural dynamics and regulatory mechanisms associated with RISC, siRNA technology has now been used to control viral infections, tumor gene modulation, metabolic diseases, and in the silencing of single genes. The use of siRNA indicates the beginning of a new era in precision, systematic approaches to RNA‐target‐specific therapeutics. siRNA mediates sequence‐specific cleavage of mRNA in a RISC‐dependent manner and is one of the conventional strategies for RNAi. Figure 3 depicts the principles and molecular mechanism of siRNA cellular delivery, target recognition and gene‐silencing activity.

### 3.2 Disease‐Targeted Applications

siRNA has become a molecular tool for precision therapy of diseases due to high specificity, efficiency, programmability, and ease of preparation. Through the selective recognition and destruction of disease‐causing gene transcripts, siRNA achieves posttranscriptional gene silencing that counters irregular protein synthesis and resets cellular signaling. Currently, siRNA‐based therapeutics have made significant transition from bench to bedside for clinical application in genetic metabolic disorders, heart disorders, cancers, and viral infections. This marks a new era in the rational and personalized development of RNA‐targeted therapeutics.

The programmed silencing of key pathogenic targets by siRNA allows multilevel regulation in the treatment of systemic diseases. For example, siRNAs designed against EGFR or KRAS could effectively inhibit the abnormal proliferation of lung cancer cells, while dual‐targeted silencing of EGFR and TNC significantly reduces glioblastoma invasiveness. In metabolic disease models, silencing of PTP1B improves insulin resistance and corrects metabolic dysregulation linked to obesity [53]. Strategies using systemically delivered siRNA represent the first of their kind to offer an entirely new approach to interventions in complex disease conditions such as cancer and the metabolic syndrome and present an innovative strategy for drug design based on the multitargeted regulation of functional pathways.

In organ‐specific therapy, the development of liver‐targeted siRNA drugs has garnered significant attention. Zodasiran reduces plasma lipid levels by inhibiting the expression of angiopoietin‐like protein 3 [54], while olpasiran decreases hepatic synthesis of lipoprotein(a), thereby significantly lowering the risk of atherosclerotic cardiovascular disease (ASCVD) [55]. RNAi‐based therapies, characterized by their high target specificity and enduring silencing effects, are emerging as innovative approaches for improving cardiac function and maintaining metabolic homeostasis [56]. Moreover, silencing of peripheral myelin protein 22 (PMP22) by siRNA shows a decrease in myelin damage and axonal injury in Charcot–Marie–Tooth disease Type 1A (CMT1A). Also, siRNA (DU01) directed at DUX4 prevents pathogenic gene expression in facioscapulohumeral muscular dystrophy, further depicting the prospective use and promise of siRNA in neurological and neuromuscular diseases [57, 58].

According to some studies, siRNA displays potent antiviral effects by either dampening the replication of viral RNA or targeting a host cofactor required for infection. RNAi tech has been effective in suppressing a virus. Studies indicate that antiviral miRNAs and siRNAs targeting foot‐and‐mouth disease virus can significantly reduce viral load and delay viral spreading, offering a molecular basis for emerging antiviral treatments of the future [59].

siRNA drugs are evolving from simple gene silencing to more complex and varied interventions. siRNA can regulate gene expression precisely, efficiently, and programmably against metabolic disorders, cancer therapy, as well as antiviral therapy. Owing to constant developments in chemical modifications, delivery system enhancements and safety evaluations, siRNA has the potential to become a cornerstone enabling technology for personalized medicine, offering new ways to intervene at the molecular level for resistant diseases.

By specifically targeting and degrading specific mRNAs, siRNAs can silence genes that have therapeutic potentials against a variety of diseases. The field of siRNA therapeutics has evolved rapidly in the last few years with several candidates that target cancers, metabolic disorders, viral infections, and rare genetic diseases making it to clinical trials. For example, single‐cell RNA sequencing has been employed to reveal targetable pathways—such as the TGF‐β signaling and proangiogenic factor secretion by mast cells—that drive heterogeneity and immunosuppression in challenging bone tumors like giant cell tumor of the bone [60]. Table 1 summarizes several target genes, their possible delivery mechanisms, and significant studies related to diverse diseases. This table will certainly help the scientists in clinical applications of RNAi‐based therapeutics.

**TABLE 1:** Targeted therapeutic applications of siRNA in various diseases.

Disease type	Target gene or pathway	siRNA delivery system	Experimental or clinical model	Therapeutic outcome	Mechanism of action	References
Hepatocellular carcinoma	MDK gene/polyamine metabolism	Dual pH‐responsive nanoparticles	Orthotopic HCC model	Inhibited tumor growth with minimal side effects	Suppressed M2 macrophage polarization and polyamine metabolism	[61]
Hepatocellular carcinoma	TGF‐β/Cox‐2 genes	Peptide nanoparticles	Homologous orthotopic HCC mouse model	Inhibited tumor growth and enhanced T‐cell infiltration	Silencing TGF‐β/Cox‐2 promotes T‐cell inflammatory activation	[62]
Hepatocellular carcinoma	MEF2D/cGAS–STING pathway	tLyp‐1‐modified liposomes	H22 cells and mouse model	Inhibited tumor growth and enhanced immunity	MEF2D knockdown reduced PD‐L1 and activated STING signaling	[63]
Hepatocellular carcinoma	CDK1 gene	TDEV membrane–hybrid lipid vesicles	Sk‐hep1 cells and tumor‐bearing mice	Inhibited tumor growth and prolonged survival	Ligand–receptor‐mediated active targeting with lysosomal escape for efficient delivery	[64]
Hepatocellular carcinoma	CD38/adenosine pathway	Extracellular vesicles	Xenograft mouse model	Inhibited tumor growth and reversed drug resistance	Reduced adenosine production and promoted M1 macrophage polarization	[65]
Hepatocellular carcinoma	Beclin 1/autophagy pathway	Calcium phosphate nanoparticles	Xenograft nude mouse model	Synergistically enhanced anticancer efficacy	Inhibited autophagy and coinduced apoptosis	[66]
Atherosclerosis	NF‐κB pathway	Tri‐component nucleic acid nanogel	Atherosclerosis model	Regressed plaques and reduced inflammation	Targeted delivery of siNF‐κB suppressed inflammation	[67]
Atherosclerosis	CaMKIIγ/MerTK pathway	NIR‐II AIE nanoparticles	Aortic plaque model	Reduced necrotic core formation	Inhibited CaMKIIγ‐mediated efferocytosis	[68]
Atherosclerosis	Wnt5a/MAPK/NF‐κB pathway	Adenoviral vector	ApoE−/− mouse model	Suppressed atherosclerosis progression	Inhibited MAPK/NF‐κB signaling pathway	[69]
Atherosclerosis	JNK2	p5RHH–siRNA nanoparticles	ApoE−/− mouse model	Reduced macrophage plaque burden and thrombosis	Inhibited JNK2 expression and downstream signaling	[70]
Neointimal hyperplasia/restenosis	Kindlin‐2/Wnt pathway	Lentiviral vector	Rat carotid balloon injury model	Inhibited neointimal hyperplasia	Suppressed Wnt/β‐catenin signaling	[71]
Atherosclerosis	Olfr2/NLRP3 inflammasome pathway	ROS‐responsive nanocarrier	Atherosclerosis model	Reduced plaque formation and inflammation	Inhibited NLRP3 inflammasome activation	[72]
MASLD	PLIN2	GalNAc–siRNA conjugate	Multiple MASLD mouse models	Improved hepatic steatosis and fibrosis	PLIN2 inhibition promoted lipid metabolism	[73]
Obesity	DPP‐4/PGC‐1α / PPARα pathway	Liver‐targeted LNPs	Diet‐induced obese mouse model	Reduced body weight and restored insulin sensitivity	Coordinated regulation of incretin secretion and thermogenesis	[74]
Obesity	SOAT2/CD36 pathway	CS–PLGA nanoparticles	Diet‐induced obese mouse model	Inhibited lipid absorption and reduced obesity	Promoted CD36 ubiquitination to reduce lipid uptake	[75]
Local adipose accumulation	TGF‐β1/COX‐2	Histidine–lysine peptide	DIO mice and miniature pigs	Reduced fat thickness and content	Silencing target genes suppressed adipogenesis	[76]
Obesity/insulin resistance	GRK2/MAPK pathway	Hydrodynamic injection	ob/ob mouse model	Improved lipid profile and liver parameters	Suppressed hepatic NEFA production pathway	[77]
Impaired wound healing in Type 2 diabetes	GM3S	Spherical nucleic acid–gold nanoparticles	Diet‐induced obese diabetic mice	Accelerated complete wound healing	siRNA‐mediated GM3S knockdown enhanced tissue repair	[78]
Breast cancer	PDGF‐D/PDGFR‐β pathway	Chitosan nanocomplexes	Breast tumor model	Inhibited tumor growth and induced apoptosis	Silenced PDGF‐D/PDGFR‐β genes	[79]
Breast cancer	17β‐HSD1	PEGylated LPD nanoparticles	T47D nude mouse model	Inhibited tumor growth	Knockdown of 17β‐HSD1 reduced estrogen production	[80]
Breast cancer	Sphk2 gene	Micelle–liposome hybrid nanoparticles	MCF‐7/ADR cells and mouse model	Inhibited tumor growth with low toxicity	Promoted apoptosis and cytotoxicity	[81]
Triple‐negative breast cancer	EGFR/BRD4 pathway	GALA/CREKA‐modified nanocarriers	MDA‐MB‐231 cell model	Inhibited proliferation, invasion, and migration	Synergistic inhibition of EGFR and BRD4 pathways	[82]
Breast cancer	VEGF	Layered intelligent nanoparticles	4T1 cells and mouse model	Inhibited tumor growth and angiogenesis	Silencing VEGF promoted apoptosis and nutrient deprivation	[83]
Triple‐negative breast cancer	IKBKE gene	Hyaluronic acid‐targeted nanocomplex	Orthotopic TNBC mouse model	Suppressed tumor proliferation and migration	Synergized with chemotherapy to enhance antitumor activity	[84]
Alzheimer's disease	BACE1	Multifunctional nanocarrier via intranasal delivery	Transgenic AD mouse model	Improved cognition and reduced deposition	Lowered BACE1, enhanced autophagy, and inhibited Aβ accumulation	[85]
Alzheimer's disease	BACE1	Glycosylated polymer nanoparticles	APP/PS1 transgenic AD mice	Improved cognitive function	Crossed BBB and silenced BACE1	[86]
Alzheimer's disease	Apoe gene	di‐siRNA GalNAc conjugate	5xFAD transgenic mice	Reduced amyloid burden and activated immunity	Silencing ApoE activated immune responses	[87]
Parkinson's disease	FTO/m6A/ATM pathway	MSC‐derived exosomes	MPTP mouse and cell models	Reduced neuronal death and restored TH expression	Inhibited FTO to suppress ATM‐mediated cell death	[88]
Parkinson's disease	LRRK2 gene	sEV‐mediated delivery	LRRK2R1441G mouse model	Ameliorated neurodegeneration and inflammation	Liver‐derived sEVs crossed BBB for delivery	[89]
Rheumatoid arthritis	NF‐κB	Folic acid‐targeted hybrid nanoparticles	Arthritis mouse model	Inhibited arthritis progression	Blocked NF‐κB signaling and reduced inflammation	[90]

Abbreviations: 5xFAD, five familial Alzheimer's disease mutations transgenic mouse model; AIE, aggregation‐induced emission; ApoE, apolipoprotein E; ApoE−/−, apolipoprotein E knockout; APP/PS1, amyloid precursor protein/presenilin 1; BACE1, beta‐secretase 1; BBB, blood–brain barrier; DIO, diet‐induced obesity; di‐siRNA, divalent siRNA; HCC, hepatocellular carcinoma; LNP, lipid nanoparticle; MASLD, metabolic dysfunction‐associated steatotic liver disease; MSC, mesenchymal stem cell; NIR‐II, second near‐infrared window; PD‐L1, programmed death‐ligand 1; PEG, polyethylene glycol; sEV, small extracellular vesicles; STING, stimulator of interferon genes; TGF‐β, transforming growth factor‐beta; TNBC, triple‐negative breast cancer; VEGF, vascular endothelial growth factor.

### 3.3 Opportunities and Challenges: Delivery Systems and off‐Target Effects

Therapeutics based on siRNA can silence a harmful gene because they recognize a particular sequence, leading to the degradation of the target mRNA. There is great potential in therapeutics based on siRNA for the treatment of metabolic, genetic, and cancerous diseases. Translating these into clinical use is challenging mainly due to two reasons. The first one is low delivery efficiency. The second one is high off‐target effect risk. Delivering siRNA efficiently and in a tissue‐targeted manner, while also achieving stable and controllable in vivo gene silencing, is an important scientific question that will determine the safe and effective use of siRNA therapeutics.

LNPs are currently the most promising clinically applicable delivery vehicles for siRNA due to their superior encapsulation efficiency, compatibility with biological systems, and scalability. Among these, GalNAc‐conjugated siRNA, which mediates liver‐specific targeting by binding to the asialoglycoprotein receptor on hepatocytes, represents a milestone. This has been successfully used in the clinic for various metabolic disorders. Yet, whether the LNPs can efficiently cross the BBB and reach the nervous system remains an important question [91, 92, 93, 94]. Furthermore, though lipid‐conjugated siRNAs may succeed in penetrating target cells more effectively, they ultimately build up in clearing organs like the liver, kidney, and spleen. For instance, an sFLT1‐targeted cholesterol‐conjugated siRNA showed delivery of approximately up to 8% of the injected dose to the placenta, although a large proportion was found in the liver and kidneys [92]. Thus, tissue selectivity and biosafety need extensive optimization.

Administering siRNA treatments that do not target the liver is highly challenging. Due to limited absorption by photoreceptor cells, the treatment of retinal degenerative disorders with siRNA may not be effective [93]. Moreover, systemic administration is associated with suboptimal gene‐silencing efficiency in different body tissues [94]. There is generally a poor uptake of siRNA by tumor cells in cancer therapy so endosomal entrapment decreases its tumor‐suppressive ability [95, 96, 97]. In addition, the delivery to the skeletal and cardiac muscles is ineffective; only very high doses (about 100 mg/kg) of cholesterol‐conjugated siRNA have shown partial uptake. However, these high amounts can cause cytokine storms and systemic inflammation [96]. These findings underscore that tissue‐specific delivery remains a critical bottleneck for siRNA therapeutics.

Immunogenicity and major off‐target effects will determine the safety profile of siRNA therapeutics. The delivery challenge is, therefore, only one of the challenges facing siRNA therapeutics. The siRNA's seed sequence may have partial complementarity with untargeted mRNAs, leading to unwanted silencing. The dsRNA structure can also activate pattern recognition receptors like TLR3, RIG‐I, and PKR, triggering the subsequent activation of interferon signaling and inflammatory pathways [95, 96, 97]. In recent decades, scientists have introduced various chemical modifications, including 2′‐O‐methylation, 2′‐fluoro substitution, and phosphorothioate linkage replacement, to improve molecular stability and reduce the recognition by the immune system. Simultaneously, bioinformatics‐guided optimization of sequences reduces undesirable seed‐region pairing with nontarget transcripts, thus improving target specificity and overall safety.

The future of siRNA therapy lies in the precision of organ‐specific LNP delivery systems. Emerging research focuses on modulating the chemical structure of ionizable lipids to achieve selective targeting of the lungs, spleen, and bone marrow. Concurrently, AI and deep learning algorithms are being employed to screen siRNA sequences that can evade immune surveillance while maximizing silencing efficiency. The integration of these technologies will drive the development of “single‐dose, long‐lasting” siRNA therapies, fundamentally altering the management of cardiovascular and metabolic chronic diseases.

## 4 Aptamer‐Based Targeted Therapy

Nucleic acid aptamers, often termed “chemical antibodies,” provide a versatile platform for high‐affinity molecular recognition and targeted delivery. In this section, we first describe the structural features and binding properties of aptamers, then summarize their disease‐targeted applications in oncology, nephrology, and immune‐mediated disorders, and finally discuss major opportunities and challenges, with particular emphasis on improving in vivo stability and controlling immunogenicity.

### 4.1 Structure and Function of Aptamers

Aptamers are short single‐stranded DNA or RNA molecules that can adopt defined three‐dimensional structures. This allows them to bind to target molecules that can be DNA, small molecule, protein, or cell surface receptors with specificity and affinity. Due to these features, aptamers are also known as “chemical antibodies.” Aptamers have several different advantages over antibodies, such as lower molecular weight, easy chemical synthesis, structural flexibility for modification, minimal immunogenicity, and reversible denaturation. These qualities make aptamers increasingly popular in fields such as RNA‐targeted therapy, drug delivery, and personalized medicine. The aptamer‐target specificity comes from the complex and stable tertiary configurations of the aptamers. These configurations include stem‐loop, hairpin, and G‐quadruplex motifs. These configurations depend on hydrogen bonds and electrostatic forces to attain a high degree of selectivity for binding to target molecules, thereby endowing aptamers with extraordinary biorecognition functionalities.

Aptamers are versatile molecular tools for effective therapy development due to their high selectivity, compatibility with biological systems, and suitability for chemical modifications. For instance, the 29‐nucleotide DNA aptamer, AJ102.29m, has been found to strongly inhibit CCL22‐dependent T‐cell migration while sparing TLR‐mediated immune activation, thus exhibiting excellent target specificity and safety [98]. Aptamers also show a higher thermal stability and structural reversibility and can be used with DNA nanotechnology for the generation of multifunctional systems with recognition, therapeutic, and diagnostic activities. For example, a hybrid nanostructure has been designed to address molecular recognition, RNA cleavage, and signaling detection with a single multifunctional nanoframe (MNF). This MNF consists of a GLUT‐1 mRNA targeted DNAzyme and a HER‐2 targeted DNA aptamer and a molecular beacon [99, 100].

Among aptamer tumor‐targeting applications, AS1411 aptamer has been one of the most studied examples. AS1411 is a single strand of guanine‐rich DNA that specifically binds to nucleolin. Nucleolin is a protein found in excessive amounts on the surface of tumor cells. In normal cells, its expression is very low. This allows AS1411 to specifically recognize and target cancer cells [101, 102]. Using this mechanism, AS1411 has been extensively employed in the development of drug delivery systems for tumors and molecular imaging probes, paving the way toward specific nucleic acid therapies. The VED–LYTAC system has significantly increased the potential applications of aptamers recently. The system utilizes three components: a motif that binds to the mannose‐6‐phosphate receptor, an RNA aptamer that targets vascular endothelial growth factor (VEGF), and a bridging linker that together induce the degradation of VEGF. This suggests that composite systems made from different aptamers hold potential for precision oncology applications [103].

**FIGURE 4:**
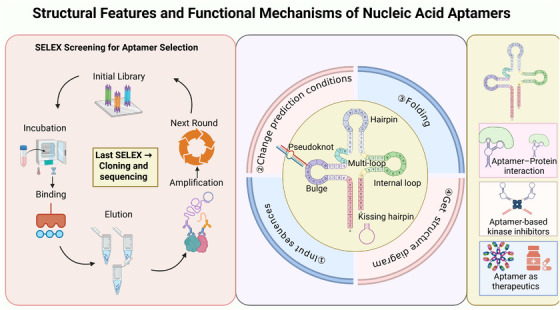
Structural properties, SELEX selection workflow, and functional mechanisms of nucleic acid aptamers. The SELEX mechanism that helps in purifying high‐affinity aptamers from a library of randomized nucleic acids is shown in the figure. Representative secondary structures such as pseudoknots, hairpins, internal loops, multiloop motifs, and kissing hairpins exhibit the patterns of folding that facilitate sequence‐specific molecular recognition. The representative examples on the right interact with protein targets, modulate kinase activity, or function as therapeutic agents. SELEX, systematic evolution of ligands by exponential enrichment.

By virtue of their programmable nucleotide sequences, tunable tertiary structures, and high‐affinity binding properties, aptamers offer an important bridge between molecular recognition and targeted therapy. As progress continues in the areas of SELEX technology, aptamer‐based DNA nanostructure engineering, and AI‐assisted molecular design, aptamers are evolving from simple recognition elements into therapeutic agents, delivery vehicles, or combined therapy‐delivery platforms. Aptamers possess the potential for a range of biomedical and therapeutic applications evidenced from the research studies. They have shown potential in RNA‐targeted therapy, precision oncology, and hybrid drug delivery systems, positioning them as a promising molecular platform for next‐generation biomedicine. Finally, aptamers fulfill their roles in molecular recognition and regulation by forming distinctive three‐dimensional structures that enable strong binding to target molecules. The structure of an aptamer and how it binds to a receptor, protein, or small molecule is depicted in Figure 4.

### 4.2 Disease‐Targeted Applications

Aptamers have unmistakably emerged as promising tools for precision therapy of several diseases because of their high affinity, remarkable target specificity, and excellent modifiability. These molecules can target and bind important proteins and can be used as inhibitors directly interfering with some abnormal signaling pathways. Alternatively, they can also serve as vehicles for drugs, nucleic acids, and nanomaterials. They can be applied in a broad spectrum as medicine for the treatment of cancer, kidney ailments, and immune‐mediated diseases.

Aptamers possess advantages in target recognition and drug delivery in oncology. The Sgc8c aptamer specifically targets protein tyrosine kinase 7 (PTK7). Numerous cancers overexpress PTK7. Studies show that it is particularly overexpressed in pancreatic cancer. The Sgc8c aptamer holds significant promise for improving treatment [104]. Likewise, a CD30‐specific aptamer‐guided multifunctional nanomedicine system can effectively target anaplastic large cell lymphoma cells in xenograft mouse models and demonstrate very good in vivo stability and targeting selectivity [105]. The aptamer chimera AS1411–ATP can detect increased intracellular ATP levels within cancer cells. Further, it is capable of triggering controlled release of doxorubicin with selective cellular uptake in cancer cells verified by flow cytometry. This strategy achieves highly specific drug delivery [106]. In another study, the E3 aptamer inhibited the growth of colorectal cancer cells from patient‐derived xenografts in vitro, and when conjugated with a toxin molecule such as MMAE or MMAF exhibited significantly improved antitumor efficacy [107]. The RLS‐2 aptamer exhibited selective distribution within the glomerular region of doxorubicin‐induced diabetic nephropathy mice in renal disease models, suggesting a benefit for kidney targeted therapy [108]. Moreover, AS1411‐conjugated gold nanoparticles showed greater antitumor efficacy than unmodified gold nanoparticles with respective IC_50_ values of 16.7 and 10.7 µg/mL. The targeting of nucleolin overexpressed on the surface of MCF‐7 cells improved cellular uptake and cytotoxic effects of the conjugates [109]. Aptamers also have potent regulatory functions in immune and inflammatory diseases. The isolated SAPT8 aptamer specifically targeting fibroblast‐like synoviocytes (FLS) of arthritic mouse models was efficiently accumulated in FLS and showed the ability to suppress inflammatory responses either alone or in the presence of anti‐TNF biologics [110]. Likewise, AptW2‐1‐39‐PEG exhibited significant anti‐inflammatory efficacy in collagen‐induced arthritis mice, leading to reduced disease score, paw swelling rate, and partially restored motor function [111].

Aptamer technology is evolving from single‐target recognition to a multifunctional, engineerable, and highly tunable therapeutic platform. The successful aims in oncology, nephrology, and immunotherapy validate its unique advantages in disease‐specific recognition and targeted regulation and provide a novel molecular basis for RNA‐targeted drug development. As we continue to see advances in chemical modification, nanocarrier engineering, and AI‐based aptamer screening, aptamers could be the essential link between molecular recognition and clinical translation for the precise treatment of complex human diseases.

### 4.3 Opportunities and Challenges: Stability and Immunogenicity Issues

Aptamers have certain advantages over antibodies including low immunogenicity, ease of screening, and low cost of production, and therefore can block target proteins. For instance, the thiophosphate‐modified RAGE aptamer shows increased stability in vivo [112]. Its strong resistance to nucleases helps retain structural and biological activity, showing great potential for drug delivery and carrier design. This feature not only significantly prolongs the circulating half‐life of aptamers and reduces the chances of rapid degradation in circulation but also enhances the therapeutic efficacy and practical utility of aptamer–drug conjugates (ApDCs) [113]. However, naked aptamers are subject to rapid nuclease degradation and have a low half‐life and poor stability in plasma. To overcome these challenges, numerous strategies involving chemical modifications have been devised to enhance aptamer stability. Techniques for enhancing resistance to exonucleases include 2′‐position modifications (e.g., fluoro, methoxy, amino groups), cyclic structures, and L‐type mirror‐image nucleic acids. Moreover, integrating aptamers into liposomes, nanoparticles or PEG‐modified delivery systems can increase circulation time and improve pharmacokinetic profiles. Significantly, studies have observed that G‐quadruplex stability can preserve a parallel conformation despite the presence of FdU at the 5′terminus [114]. In addition, the covalent conjugation of aptamers to therapeutic molecules can prevent hydrolysis by nucleases and rapid clearance by the kidneys, leading to clinical translation [115].

Aptamers also have an immunogenicity issue that limits their clinical use. Aptamers tend to have a lower immunogenicity than antibodies derived from proteins, but some CpG‐enriched aptamers are capable of TLR9 activation and subsequent cytokine release and inflammation. To avoid immune recognition, beneficial strategies include modification of CpG sequences, incorporation of unnatural nucleobases, or use of D‐form nucleic acids. Besides, conjugation of aptamers with nanocarriers may alter the surface charge and spatial conformation. This can impact their immunocompatibility, and thus proper immunotoxicological evaluation is required. In summary, chemical modification is an important prerequisite for improving the metabolic stability, cellular uptake, and bioactivity of oligonucleotide‐based therapeutics like ASOs, RNAi molecules, and aptamers [116].

As chemical modification technology, delivery vector engineering, and AI‐assisted screening technology develop rapidly, aptamer stability and immunological safety issues will be systematically resolved. Aptamers, as promising innovative bioactive agents for active‐targeted cancer therapy, have advantages such as high affinity, excellent tissue penetration, low immunogenicity, ease of mass production, batch‐to‐batch consistency, and long shelf life [117].

For aptamer technology, AI‐driven 3D structure prediction and high‐throughput microfluidic SELEX techniques are expanding its translational potential. These tools accelerate the discovery of “smart aptamers” that undergo conformational changes in response to specific tumor microenvironments. Additionally, the development of ApDCs represents a powerful alternative to ApDCs, offering superior tissue penetration and reduced immunogenicity. Future efforts will focus on establishing standardized chemical modification strategies to ensure clinical‐grade stability and consistent pharmacokinetics.

## 5 mRNA‐Based Targeted Therapy and Vaccine Applications

mRNA therapeutics have rapidly evolved from an experimental concept into a clinically validated platform for protein replacement and vaccination. This section begins by outlining the core mechanisms and unique advantages of mRNA‐based therapeutics, then highlights representative disease‐targeted applications in cardiometabolic, inflammatory, pulmonary, and oncologic settings, and finally discusses key opportunities and challenges related to transcription/translation efficiency and the fine‐tuning of immune responses.

### 5.1 Mechanisms and Advantages of mRNA Therapeutics

mRNA therapeutics are new types of medicine that use the body's cells as biological factories. These drugs involve the introduction of synthetic mRNA molecules that, when delivered into cells, lead to ribosome‐mediated translational synthesis of functional proteins in the cytoplasm. This process can treat and prevent diseases by the restoration of protein deficiencies due to genetic mutations [118]. Unlike traditional gene therapy, mRNA therapeutics work only in the cytoplasm, eliminating the need to enter the nucleus, thus lowering the risks associated with genomic integration and insertional mutagenesis [119]. mRNA‐based therapies have been successfully employed for protein replacement therapy, immune activation, and tissue regeneration to date. Gene editing has shown remarkable potential for tackling genetic disorders, treating infectious diseases, and improving cancer immunotherapy [120, 121, 122].

Therapeutic mRNAs are generally produced by in vitro transcription and packaged into LNPs to facilitate cellular delivery. Modified nucleotides like N1‐methylpseudouridine are incorporated into mRNA to enhance molecular stability and translation efficiency and to reduce innate immune activation [123]. For example, the STLNPs–Man@mRNAOVA system displayed a similar antitumor effect at one‐fifth the dose of a classical vaccine but significantly reduced immune‐related adverse events [124], providing experimental evidence for the safety and efficacy of optimized mRNA vaccine platforms.

mRNA therapeutics offer many significant advantages over DNA gene therapies: they do not depend on nuclear localization, directly express proteins in the cytoplasm, elicit predictable expression kinetics, and are fully biodegradable. Taken together, these features add to their safety and control [119, 120, 121, 122, 125]. Moreover, mRNA's adaptable and programmable nature permits speedy alterations through changes to the coding sequence, allowing for the quick development of personalized cancer vaccines and precision therapies for rare diseases [121]. Lately, the engineering of in vivo mRNA‐based chimeric antigen receptor T‐cells has gained great prominence in research. Unlike traditional ex vivo modification methods, this approach achieves significant simplification of the manufacturing process while allowing for transient and controllable gene expression, thereby reducing any safety concerns associated with constitutive modification [126].

**FIGURE 5:**
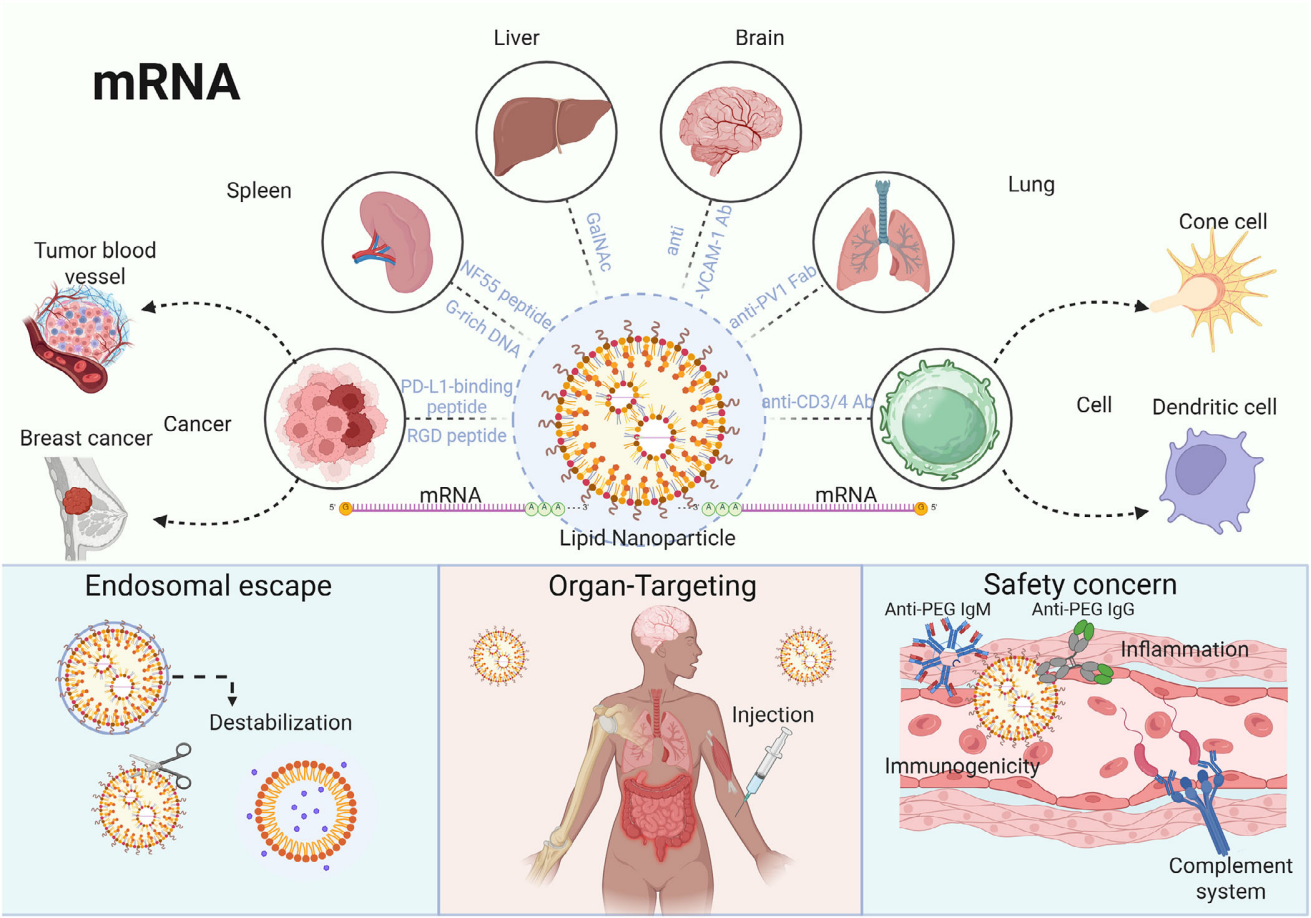
Key components, targeting strategies, and safety considerations of mRNA–LNP delivery systems. The structural features of the mRNA‐loaded LNPs are shown in the figure, and the surface ligands such as peptides, antibodies, or glycan modifications help direct organ‐ or cell‐specific delivery to the liver, spleen, lungs, brain, and tumors. Once cells take up LNPs, the particles disintegrate within endosomes, releasing mRNA into the cytoplasm where it is translated. Potential safety concerns are also highlighted, including PEG‐related immunogenicity, complement activation, and inflammatory reactions. mRNA, messenger RNA; LNP, lipid nanoparticle; PEG, polyethylene glycol.

mRNA technology, which belongs to the family of next‐generation vaccines, possesses intrinsic adjuvant properties, low production costs, and rapid redesign of antigen epitopes. Thus, mRNA vaccines possess unique advantages for responding to emerging infectious diseases and tumor immunotherapy [127]. Overall, mRNA therapeutics are emerging as a rapidly growing core technology of precision medicine and molecular therapeutics due to their high efficiency, safety, flexibility, and personalized adaptability. mRNA‐focused therapies not only replace proteins but also, through in situ translation of functional proteins, help to activate the immune system. This approach provides a unique theoretical and technical foundation to tackle complex human diseases. Figure 5 illustrates the delivery of mRNA therapeutics into cells and their subsequent cellular impact. It also highlights the biological benefits of mRNA therapy, which has a dual function: providing both a functional protein and an immune modulator. This allows for greater flexibility in therapeutic applications compared with other treatments.

### 5.2 Disease‐Targeted Applications

The rapid advancement of mRNA therapeutics has exposed the vast potential of mRNA for the precise regulation of different diseases. Through the synthetic generation and delivery of functional mRNA, therapeutic agents may be designed to elicit controlled in vivo production of specific proteins, allowing for the modulation of key molecular pathways involved in genetic disorders, cancer, infectious disease, and immune‐mediated disease. mRNA is programmable, and by teaming it with efficient delivery technologies, it can serve as an important bridge between molecules and clinics.

mRNA‐based therapies have made great progress in metabolic and cardiovascular diseases. Researchers have utilized a liver‐specific SORT LNP system for the codelivery of Cas9 mRNA and sgRNA targeting PCSK9 to achieve an almost complete (∼100%) knockout of the PCSK9 gene, resulting in a marked decrease in serum PCSK9 levels. This provides a precise molecular intervention strategy for hypercholesterolemia and ASCVDs [128]. Similar approaches have been utilized for inherited metabolic disorders like methylmalonic acidemia and cystic fibrosis delivery of mRNAs coding functional enzymes restores metabolic homeostasis and improves organ function, improves organ function, and has long‐term potential for therapeutic protein replacement by mRNA.

The effectiveness of mRNA therapeutics in adjusting immune homeostasis is evidenced in inflammatory and autoimmune diseases. The intestinal delivery of nanoparticles encapsulating IL‐10 mRNA significantly reduced the levels of proinflammatory cytokines in systemic circulation and tissue in colitis models, thereby alleviating both acute and chronic intestinal inflammation [129]. In animal models of multiple sclerosis and Type 1 diabetes, administering mRNAs encoding antigens that induce immune tolerance led to expansion of regulatory T cells and reestablishment of immune balance. Thus, this represents a new nonviral molecular therapy for autoimmune diseases.

Even in rare and structural lung diseases, mRNA technology has shown great therapeutic promise. LNP‐based mRNA delivery systems efficiently target the lungs in LAM mouse models. Delivery of Tsc2 mRNA restored TSC2 tumor suppressor function, greatly reducing tumor burden and improving lung function [130]. The TD5 BLNP formulation, when administered intrathecally, likewise delivered mRNA to the CNS with neurons and astrocytes showing high expression. This intervention represents a promising therapeutic approach for reparative interventions in neurodegenerative diseases and brain injuries [131].

In cancer immunotherapy mRNA platforms have shown great versatility. Lipid–polymer particle‐formulated mRNA vaccines have generated strong, neoantigen‐specific CD8^+^ T cell responses and inhibited tumor growth in a number of murine tumor models, including CT26, MC38, and B16F10 [132]. The codelivery of mRNA with guide RNA enhances delivery efficiency of CRISPR/Cas9 and is a useful strategy for targeting metastatic tumors like breast carcinoma with genetic therapies [133]. The structure‐anchored targeted RNA delivery platform has allowed locally and lung‐targeted nanoparticles (LNPLocal and LNPLung) to efficiently deliver IL‐15 superagonist mRNA, which activates the immune microenvironment within the local tumor and augments systemic antitumor immunity [134]. What is more, the mLPR vaccine was able to inhibit lung cancer growth and reduce bone metastasis by promoting the release of IFN‐γ and IL‐12, as well as enhancing natural killer cell‐mediated antibody‐dependent cytotoxicity [135].

In the field of viral infections, the most representative clinical translation of mRNA technology has come from vaccine development during the COVID‐19 pandemic. Several clinical studies have shown that mRNA–LNP vaccines can rapidly induce robust humoral and cellular immune responses, demonstrating strong protection even in immunocompromised populations, such as the elderly and cancer patients. For example, innovative mRNA vaccine technology, through a dual antigen presentation mechanism on both cell surfaces and extracellular particles, can induce more potent B cell activation and antibody responses compared with traditional sequential immunization, significantly enhancing the breadth and durability of immune defense against SARS‐CoV‐2 variants like Omicron [136]. Additionally, the rapid deployment of the XBB.1.5 mRNA booster strongly supports the strategic value of the mRNA vaccine platform in enabling rapid antigen iteration and responding to high‐mutagenic pathogens, while also highlighting the maturity and scalability of this technology in establishing robust systemic immune defenses [137]. The continuous evolutionary pressure, as detailed by studies on critical Omicron spike mutations and transmission drivers, underscores the necessity for this rapid‐response platform [138].

Through mRNA therapeutics designed to reprogram networks of intracellular protein expression, multifaceted interventions such as gene editing, immune modulation, and tissue regeneration are facilitated. The success of mRNA‐based approaches in the management of cardiometabolic diseases, colitis, rare pulmonary disorders, and cancer therapy shows a broad therapeutic scope and significant translational potential for RNA‐based medicine. Together, these advances lay a solid foundation for the clinical translation and precise development of mRNA therapeutics. mRNA therapy, through mediating in vivo translation of functional proteins or antigens, is a powerful agent to prevent and treat diseases. They have many applications in infectious diseases, cancer, and genetic diseases. Their applications for each disease type, including molecular target, delivery system, and clinical development status, are systematically summarized in Table 2, serving as a comprehensive reference for the design and translational development of mRNA‐based therapeutics.

**TABLE 2:** Targeted therapeutic and vaccine applications of mRNA in various diseases.

Disease type	Target gene or antigen	Delivery system	Activated immune/signaling pathway	Primary therapeutic effect	Mechanism of action	References
Pancreatic ductal adenocarcinoma	Neoantigen	LNP	Neoantigen‐specific CD8^+^ T cells	Associated with delayed relapse	Activation/amplification of neoantigen‐specific T cells	[139]
Melanoma (cancer)	OVA model antigen, TRP‐2 peptide	113‐O12B LNP	CD8^+^ T cell response	Tumor suppression, complete remission and long‐term immune memory	Lymph node‐targeted delivery of mRNA to enhance immunity	[140]
HPV‐associated malignancies	HPV16/18 E2, E6, E7 antigens	LNP	CD8^+^ T cell response	Tumor regression and generation of long‐term immune memory	Induction of antigen‐specific T cell immunity	[141]
Clostridioides difficile infection	Toxin, virulence factors and spore antigens	LNP	Humoral and cellular immune responses	Prevention of lethal infection and eradication of bacterial colonization	Induction of antigen‐specific immune responses	[142]
Melanoma and colorectal cancer	Tumor antigen	Spleen‐targeted Mn@mRNA–LNP	STING pathway and Type I interferons	Inhibition of tumor progression, achieving complete suppression	Spleen‐targeted delivery and synergistic immune activation	[143]
Pancreatic ductal adenocarcinoma	Neoantigens (somatic mutation‐derived)	mRNA–LNP	CD8^+^ T cell response	Prolonged relapse‐free survival	Induction of newly formed long‐lived CD8^+^ T cells	[144]
Metastatic gastrointestinal cancer	Neoantigens and KRAS G12D mutation	mRNA constructs	Mutation‐specific T cell response	Good safety profile, no objective clinical responses observed	Induction of neoantigen‐specific T cells	[145]
Lyme disease	Outer surface protein A (OspA)	LNP	Humoral and cellular immune responses	Prevention of bacterial infection	Induction of humoral and cellular immunity	[146]
COVID‐19 (caused by SARS‐CoV‐2)	SARS‐CoV‐2 spike protein	PACE polymer	Cellular and humoral adaptive immunity	Protection against lethal viral infection	Lung‐targeted delivery of mRNA to elicit immunity	[147]
Pulmonary disorders (e.g., tumors, inflammation)	Model protein (e.g., luciferase)	Dual‐targeted mRNA nanoparticles	Delivery efficiency and protein expression	Efficient targeted protein expression in lung	Dual‐targeted delivery to specific lung cells	[148]
HIV	N332–GT5 immunogen and BG18 antibody	mRNA–LNP	Germinal‐center B cell response	Induction of B cell responses and affinity maturation	Driving B cell maturation through mRNA delivery	[149]
Triple‐negative breast cancer	METTL16 gene and mMUC1 antigen	LNP	Activation of antitumor immune responses	Inhibition of tumor growth and lung metastasis	Silencing METTL16 combined with vaccine immunization	[150]
SARS‐CoV‐2 infection	SARS‐CoV‐2 spike protein	PFS nanoparticles	Humoral immune response	Neutralization of SARS‐CoV‐2 virus	Enhanced cellular uptake and endosomal escape	[151]
Multiple myeloma	CT7, MAGE‐A3, WT1	mRNA electroporated dendritic cells	T cell immune responses	Safe and immunogenic	Antigen‐loaded dendritic cells activate T cells	[152]
COVID‐19	SARS‐CoV‐2 spike protein RBD	BNT162b2 vaccine	Plasmablast and antibody responses	Induces B cell response, but antibodies tend to wane	Encoding spike protein to elicit humoral immunity	[153]
Lung cancer and virus‐induced lung injury	Gene‐editing targets and angiogenic factors	Siloxane‐LNPs	Activation of angiogenic pathways	Robust gene knockout and lung injury recovery	Enhanced cellular internalization and endosomal escape	[154]
Influenza A H1N1	Influenza A H1N1 hemagglutinin	Mannose–chitosan LNP	Humoral and cellular immune responses	Generation of neutralizing antibodies and mucosal immunity	Intranasal delivery inducing systemic and mucosal immunity	[155]
COVID‐19	SARS‐CoV‐2 spike protein RBD	mRNA vaccine (study subject)	Memory B cell and antibody responses	Comparable neutralizing potency but attenuated immune response	Induction of humoral immunity and memory B cells	[156]
Colorectal cancer and melanoma	Ovalbumin (model antigen)	MO@NAL nanoparticles	Neutralization of tumor microenvironment acidity and enhancement of T cell response	Suppression of tumor growth and progression	Antigen labeling and immunomodulation within the tumor microenvironment	[157]
Cancer (E.G7‐OVA tumor model)	Ovalbumin (model antigen)	O12‐Tta‐CDs carbon‐dot nanoparticles	Dendritic cell and T cell immune responses	Tumor growth inhibition and relapse prevention	Spleen‐targeted delivery promoting antigen presentation	[158]
Prostate cancer	5T4 and CD70	LNP	Humoral and cellular immunity (CD8^+^ T, NK cells)	Tumor inhibition and prolonged survival	Dual‐antigen mRNA synergistically activating immune response	[159]
Influenza B	Neuraminidase and M2e	LNP	Humoral and cellular immune responses	Broad‐spectrum viral protection	Encoding novel target antigens to synergistically enhance immunity	[160]
Melanoma	Gp‐100 antigen	Cationic liposomes GD‐LPR	TLR7 and PERK pathways, enhanced immunity	Tumor growth and metastasis inhibition	Co‐delivery modulating the tumor microenvironment	[161]
Fish bacterial disease (Vibrio anguillarum infection)	circRNA circMIB2/protein MIB2‐134aa	Endogenous circRNA study	TRAF6 K11 ubiquitination and innate immunity	Inhibition of pathogen colonization in vivo	Coding protein regulates TRAF6 ubiquitination	[162]
COVID‐19	SARS‐CoV‐2 variant spike protein	Variant‐targeted mRNA vaccines	Humoral immunity (focus on protective efficacy)	Short‐term prevention of severe disease and hospitalization	Encoding variant antigens to enhance immune protection	[163]
Cancer patients prophylaxis for COVID‐19	SARS‐CoV‐2 spike protein	mRNA vaccine	Humoral and T cell immune responses	Prevention of severe disease, mild breakthrough infections	Activation of immunity generating antibodies and T cells	[164]
Cancer patients under active treatment	SARS‐CoV‐2 spike protein (BNT162b2 mRNA vaccine)	LNP‐encapsulated mRNA vaccine	Humoral immune response (IgG seroconversion)	Reduced seroconversion rates in patients receiving chemotherapy, hormone therapy, or targeted therapy; mild adverse events	Induction of antigen‐specific humoral immunity; reduced vaccine efficacy associated with immunosuppressive treatments and low lymphocyte counts	[165]

Abbreviations: CD8+, cluster of differentiation 8 positive; circRNA, circular RNA; COVID‐19, coronavirus disease 2019; HIV, human immunodeficiency virus; HPV, human papillomavirus; IgG, immunoglobulin G; LNP, lipid nanoparticle; mRNA, messenger RNA; NK, natural killer; OVA, ovalbumin; PACE, poly(amine‐co‐ester); PERK, protein kinase R‐like endoplasmic reticulum kinase; RBD, receptor‐binding domain; SARS‐CoV‐2, severe acute respiratory syndrome coronavirus 2; STING, stimulator of interferon genes; TLR7, toll‐like receptor 7.

### 5.3 Opportunities and Challenges: Transcription Efficiency and Immune Response

mRNA‐based therapies have made significant strides in vaccine development and disease treatment. Nonetheless, their clinical translation is still hampered by two main concerns: low translation efficiency and the need for precise immune response regulation. mRNA molecules are prone to nuclease degradation and activation of innate immune sensors in vivo, prompting researchers to optimize stability, translation efficiency, and immune controllability to refine RNA therapeutics. Studies show that incorporation of all‐trans retinoic acid into LNPs greatly enhances mRNA transfection efficiency and T‐cell upregulation of gut‐homing receptors, which augments vaccine‐induced immune responses [166]. Furthermore, the covalently closed‐loop configuration of circRNAs confers enhanced molecular stability, leading to prolonged protein expression and yields that are several hundred times greater than those from linear mRNAs [167]. CircRNAs are also highly effective at evading immune detection in many tissues, circumventing recognition by the innate immune system without the need for nucleoside modification. This property showcases the potential of circRNAs as vehicles for the expression of therapeutic proteins [168].

LNPs play a key role in protecting mRNA from degradation by enzymes and facilitating cell uptake. Furthermore, they exhibit the capacity to stimulate robust, antigen‐specific immune responses and have adjuvant‐like properties [169]. However, excessive immune activation may cause localized or systemic inflammation, which raises safety issues. Consequently, it is important to achieve a proper balance between immunostimulation and biocompatibility via precise tuning of the ionizable lipid ratios and surface structural engineering [170]. In addition, epitranscriptomic modifications on RNA molecules strongly influence immune recognition and translation. Aberrant redistribution and activation of m^6^A modifications on pseudogene‐derived lncRNA transcripts are observed in high‐grade serous ovarian cancer. In particular, the m6A‐regulated RPS15AP12–lncRNA binds miR‐96‐3p as a competing endogenous RNA to repress innate immunity and accelerate tumorigenesis [171]. Likewise, the G332A alteration within nsp14 slows viral replication, increases immune sensing, and lowers efficiency of RNA translation [172]. Viruses exploit RNA methylation to achieve both immune‐evasive and immune‐stimulatory effects. Scientific investigations reveal that methylation of antiviral transcripts is promoted by RNA helicase DDX5, which augments viral replication and spread. DDX5 therefore acts as an inhibitory regulator of innate immunity, which assists viruses in evading the immune responses of the host [173]. According to these findings, RNA modifications can be used to decrease the immunogenicity of therapeutic mRNAs, but they can also be used by pathogens to evade host immunity.

Achieving a balance between translational efficiency and immune regulation for mRNAs and their derivatives (e.g., circRNAs and modified lncRNAs) is pivotal for the future of RNA‐based therapeutics. Emerging studies should focus on the combined development/optimization of highly‐stable nucleoside modifications, precise LNP designs, and tailor‐made immune modulation strategies. This approach aims to attain a dynamic balance that enables high expression levels while maintaining low immunogenicity. The development will enhance the safety and efficacy profile of mRNA therapies in various disease settings for clinical use.

The evolution of mRNA therapies is rapidly advancing toward circRNA vaccines, which offer superior stability and more prolonged protein expression compared with linear mRNA. Alongside this, optimization of organ‐specific LNP delivery technologies is underway, with high‐throughput screening methods identifying lipid formulations capable of crossing biological barriers, such as the BBB. Additionally, the application of AI in codon optimization and untranslated region (UTR) design will further enhance translation efficiency and modulate immunogenicity, paving the way for next‐generation personalized cancer vaccines and protein replacement therapies.

## 6 miRNA‐Based Targeted Therapy

miRNAs are key posttranscriptional regulators that fine‐tune gene expression networks and are deeply involved in metabolic, cardiovascular, and inflammatory pathologies. In this section, we first summarize the biogenesis and molecular functions of miRNAs, then review their roles in disease regulation and miRNA‐directed therapeutic strategies, and finally discuss opportunities and challenges centered on target specificity, biodistribution, and delivery optimization.

### 6.1 Biological Characteristics of miRNAs

miRNAs are a class of endogenous noncoding RNAs that are approximately 20–24 nucleotides long. miRNAs regulate gene expression by posttranscriptionally silencing target mRNAs in eukaryotic cells. miRNAs repress the protein coding capability of target mRNAs by inducing their degradation and/or inhibiting their translation. Moreover, miRNAs have been found to regulate various biological processes such as cell proliferation, differentiation, apoptosis, metabolic homeostasis, and immunity [174, 175, 176]. The principal function of miRNAs at a molecular level is mainly through their interaction with AGO proteins allowing the RISC assembly. This complex binds to the 3′UTR of target mRNAs to initiate posttranscriptional gene silencing, which is the mechanism of action of miRNAs [177, 178, 179].

The evolutionarily conserved process that gives rise to miRNAs is carried out in multiple steps. This involves steps including transcription inside the nucleus, enzymatic processing, and maturation in the cytoplasm. The precursor miRNAs (pri‐miRNAs) are made from the initial transcription of these genes by RNA polymerase II. This transcript is then cleaved by the Drosha–DGCR8 microprocessor complex to generate pre‐miRNAs that are exported to the cytoplasm (with the help of exportin‐5). In the cytoplasm, Dicer, an RNase III endonuclease, processes the pre‐miRNA into a miRNA duplex of ∼22 nucleotides. The guide strand of the duplex is selectively loaded into RISC to create a mature and functional miRNA. The complementary passenger strand is destroyed in the process [178, 179]. The seed sequence of a miRNA, usually 2–8 nucleotides at the 5′ end, is important for target recognition. The complementarity between the seed region and the target mRNA sequence determines the specificity and efficiency of regulation [179, 180, 181]. Gene expression can be modulated by miRNAs via mRNA cleavage, repression of translation, and mRNA decay through deadenylations [181]. A miRNA can regulate multiple target genes at once. Likewise, one gene can be coregulated by multiple miRNAs. This often leads to complex regulatory networks. The miRNAs are regulated at multiple levels and can have an important role in maintaining cellular homeostasis and responding to environmental factors. miRNA expression has spatiotemporal specificity as it varies according to tissue type, developmental stage, cell function, and changes dynamically when affected by external or pathological factors. The highly specific expression patterns of miRNAs highlight their vital role in maintaining tissue homeostasis, determination of the cell fate, and disease progression. Extensive studies show a strong correlation between abnormal miRNA expression and disease. Dysregulation, which can involve upregulation or downregulation, may lead to cellular dysfunction, metabolic imbalance, or immune abnormality. Alterations in miRNA expression levels and their specific tissue distribution provide a molecular basis for early disease diagnosis, classification, and therapy.

**FIGURE 6:**
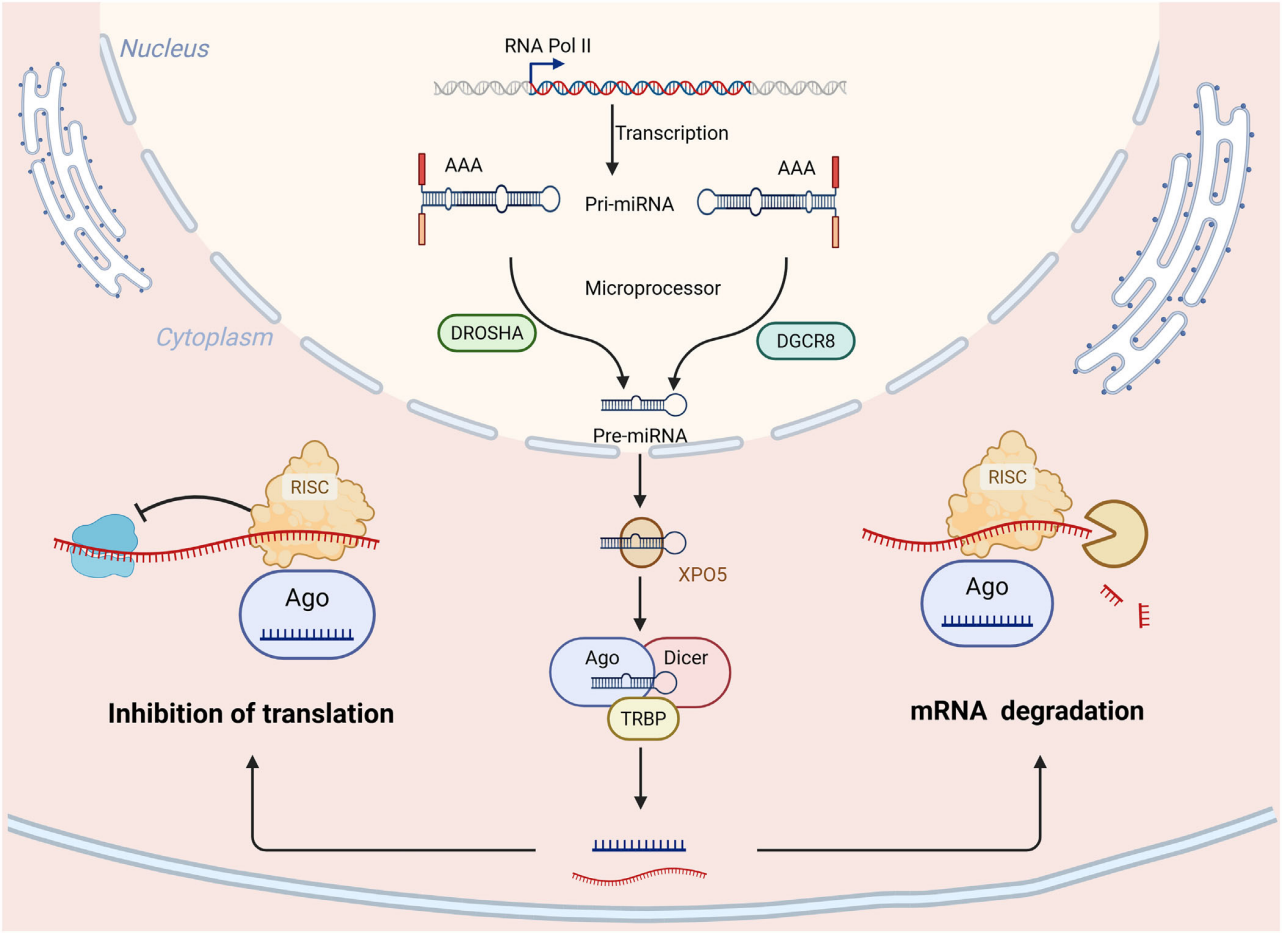
Biogenesis of miRNAs and their RISC‐mediated posttranscriptional regulatory mechanisms. The diagram shows the transcription of pri‐miRNAs by RNA polymerase II, followed by their processing into pre‐miRNAs by the Drosha–DGCR8 complex and subsequent export via exportin‐5. In the cytoplasm, the pre‐miRNA is processed by Dicer (in complex with TRBP) into mature miRNA duplexes, after which the guide strand is incorporated into an AGO‐containing RISC complex. The mature RISC complex then binds to target mRNAs via sequence complementarity, mediating their translational inhibition or degradation. miRNA, microRNA; pri‐miRNA, primary microRNA; pre‐miRNA, precursor microRNA; RISC, RNA‐induced silencing complex; AGO, argonaute; DGCR8, DiGeorge syndrome critical region gene 8; TRBP, TAR RNA‐binding protein; mRNA, messenger RNA.

As core regulators of posttranscriptional gene expression in eukaryotic cells, miRNAs function through RISC‐mediated mRNA degradation and translational inhibition, thereby maintaining cellular equilibrium and modulating pathophysiological processes [174, 175, 176, 177, 178, 179, 180, 181]. The significant conservation of their biological functions and their extensive regulatory capabilities not only unveil a further intricate layer in the regulation of genes but also form a solid theoretical basis for the advancement and clinical use of RNA‐targeted therapies. The biogenesis pathway of miRNAs and the molecular processes by which they influence posttranscriptional gene regulation are depicted in Figure 6.

### 6.2 miRNA‐Mediated Disease Regulation and Targeted Therapy

Research has elucidated the role of miRNAs in metabolic and cardiovascular diseases. By controlling key cellular processes such as energy metabolism, lipid storage, immune response, and apoptosis, miRNAs contribute to abnormal structural changes in injuries. According to the available evidence, altered miRNA expression correlates with diseases such as diabetes mellitus, NAFLD, atherosclerosis, and heart failure. Because of their ability to finely regulate gene activity after transcription, miRNA‐based interventions are promising for precision therapy of metabolic and cardiovascular diseases.

In metabolic disorders, miRNAs are important for maintaining energy balance and metabolic homeostasis through the modulation of insulin signaling, lipid metabolism, and liver fibrogenesis. Research has demonstrated that deficient levels of miR‐22 can stimulate energy expenditure and facilitate white adipose tissue browning, which may be exploited in therapeutic approaches against obesity and metabolic‐associated disorders [182]. Inhibition of miR‐22‐3p significantly ameliorates insulin resistance and alleviates hepatic steatosis, exerting beneficial effects in diabetes and NAFLD [183]. Moreover, combinatorial strategies that combine pharmacological modulation with miRNA regulation are delivering encouraging results. To illustrate, the SGLT2 inhibitor empagliflozin has been shown to alleviate hepatic fibrosis associated with NAFLD by inhibiting the expression of miR‐34a‐5p, while also enhancing the expression of its target gene, GREM2. So, the TGF‐β signaling pathway is inhibited accordingly. This demonstrates the involvement of miRNA mechanisms in metabolic remodeling [184].

In cardiovascular diseases, miRNAs play important roles in regulating cardiac remodeling, inflammation, and the progression of atherosclerosis. Macrophage‐exosomal miR‐21‐3p promotes VSMC migration and proliferation by targeting PTEN, which accelerates atherosclerotic plaque development [185]. The downregulation of miR‐199a‐3p and miR‐15a‐5p leads to NF‐κB pathway activation and enhanced inflammatory response [186]. Furthermore, potential therapeutic targets against atherosclerosis include miR‐320b, miR‐127‐3p, and miR‐299‐3p, which are associated with cholesterol efflux, lipid metabolism regulation, and plaque stability maintenance, respectively [187, 188, 189]. In heart failure cases, circulating miR‐21 levels have a positive relation with levels of inflammatory cytokines. This relationship reflects the systemic inflammatory burden, suggesting miR‐21 is a potential prognostic biomarker as well as therapeutic target [190]. At the same time, fibroblasts‐derived miR‐425‐5p alleviates cardiac injury caused by pressure overload by inhibiting myocardial fibrosis and remodeling. This shows that fibroblast‐derived miR‐425‐5p possesses a dual diagnostic and therapeutic potential [191].

miRNAs can either exacerbate or ameliorate disease conditions. This dual role is evident in both metabolic and cardiovascular pathophysiology. Using ASOs, LNA‐modified anti‐miRs, miRNA mimics, and other molecular approaches enables precise modulation of key miRNAs and the rewiring of metabolic and cardiovascular signaling networks at the molecular level [184, 185, 186, 187, 188, 189, 190, 191]. It is expected that therapeutics targeting miRNAs will overcome some of the shortcomings of traditional pharmacotherapy as the chemistry of RNA modifications improves and delivery systems advance. These therapies offer highly specific and enduring molecular interventions for complex, multigenic diseases. By affecting gene expression posttranscription, miRNAs are important for metabolic and cardiovascular function. Recent studies show that miRNAs are potential therapeutic targets for metabolic and cardiovascular diseases. Further research findings on miRNA targeted therapy for metabolic and cardiovascular diseases are summarized in Table 3. The table offers in‐depth details about significant molecular targets, signaling pathways, and therapeutic approaches. Thus, it provides a comprehensive framework that underlines the advantages of miRNA modulation for treating chronic diseases.

**TABLE 3:** Targeted therapeutic applications of miRNAs in metabolic and cardiovascular diseases.

Disease type	Target miRNA	Target gene/pathway	Intervention	Molecular mechanism	Therapeutic effect	References
Hypertension	miR‐181a	Renin–angiotensin system	Intravenous administration of miR‐181a mimics	Upregulation of miR‐181a suppresses RAS activation	Reduces blood pressure	[192]
Hypertensive vascular sclerosis	miR‐214	T‐cell activation/chemotaxis pathway	miR‐214 gene knockout	Regulates T‐cell recruitment and cytokine secretion	Attenuates perivascular fibrosis	[193]
Hypertension	rno‐miR‐126a‐5p/miR‐31a	Dbp/Crot/Mrpl4	Small‐molecule agents and miRNA modulation	Regulation of target gene expression	Potential therapeutic targets for hypertension improvement	[194]
Pulmonary hypertension	miR‐124	Chromatin remodeling/epigenetic regulation	HDAC inhibitor or CRISPR–dCas9–HAT system	Regulates miR‐124 transcription and epigenetic modification	Reverses pulmonary vascular cell activation	[195]
Pulmonary arterial hypertension	miR‐199a‐5p	Smad3 signaling pathway	Anti‐miR‐199a‐5p inhibitor	Modulates Smad3‐mediated signaling	Reduces pulmonary artery pressure and hypertrophy	[196]
Stress‐induced hypertension	miR‐335/miR‐674‐3p	Sphk1/apoptosis axis	Intracerebral injection of miR modulators	Inhibits Sphk1‐driven apoptotic signaling	Lowers blood pressure and heart rate	[197]
Diabetic nephropathy	miR‐146a‐5p	TRAF6/STAT1 signaling pathway	Umbilical cord‐derived MSCs and exosomes	Inhibits TRAF6 to promote M2 macrophage polarization	Improves renal function and reduces inflammation	[198]
Diastolic dysfunction/HFpEF	miR‐30d‐5p/miR‐30e‐5p	eNOS/fatty acid metabolism pathway	Knockdown of miR‐30 family members	Enhances oxidative stress and inhibits eNOS signaling	Improves microvascular dysfunction	[199]
NAFLD	miR‐32‐5p	INSIG1/SREBP pathway	miR‐32 antagonist	Inhibits INSIG1 activation of SREBP	Attenuates hepatic steatosis	[200]
Hyperlipidemia	miR‐146a/miR‐142a	NF‐κB/GLUT1 and CPT1A	miRNA mimics/antagonists	Regulates glycolysis and oxidative phosphorylation	Suppresses inflammation and hematopoiesis	[201]
Hyperlipidemia	miR‐145‐5p	PAK7/β‐catenin pathway	Regulation of miR‐145‐5p	Targets PAK7 to promote M2 polarization	Improves lipid metabolism and macrophage polarization	[202]
Hyperlipidemia	miR‐185‐3p	MAML1	Adenoviral vector overexpression	Targets and inhibits MAML1 expression	Improves lipid profile and alleviates liver injury	[203]
Hyperlipidemia	miR‐378a‐3p	Sort1/ApoB100 pathway	miR‐378a‐3p inhibitor	Inhibits Sort1 to reduce VLDL secretion	Lowers VLDL/LDL and triglycerides	[204]
Acute myocardial infarction	miR‐139‐3p	Stat1 signaling pathway	MSC exosome injection	Inhibits Stat1 to promote M2 polarization	Improves cardiac function and reduces infarct size	[205]
Myocardial infarction/I‐R injury	miR‐302	Hippo/Yap pathway	Engineered exosome delivery	Activates Yap to promote cell proliferation	Improves cardiac function and reduces infarction	[206]
Acute myocardial infarction	miR‐450b‐5p	ACSL4	Silencing NEAT1	ceRNA mechanism inhibits ferroptosis	Attenuates myocardial injury	[207]
Acute myocardial infarction	miR‐145	Akt3/mTOR pathway	miR‐145 overexpression	Promotes autophagy and inhibits apoptosis	Reduces cardiomyocyte apoptosis	[208]
Acute myocardial infarction	miR‐205	miR‐205 signaling pathway	ADSC exosome injection	Inhibits apoptosis and promotes angiogenesis	Improves cardiac function and reduces damage	[209]
Heart failure	miR‐182	PDCD4/PACS2	AAV vector injection	Downregulates PDCD4/PACS2 to suppress apoptosis	Improves cardiac function and reduces apoptosis	[210]
Heart failure	miR‐129‐5p	ETS2/TUG1/ATG7 axis	ETS2 silencing	ceRNA mechanism suppresses autophagy and apoptosis	Slows progression of heart failure	[211]
Heart failure/bradyarrhythmia	miR‐370‐3p	HCN4 ion channel	Intraperitoneal injection of anti‐miR	Inhibits HCN4 to prevent bradycardia	Restores heart rate and cardiac function	[212]
Heart failure	miR‐425‐5p	TGF‐β1/Smad signaling pathway	AAV‐mediated overexpression	Targets TGF‐β1/Smad pathway	Reduces fibrosis and improves cardiac function	[191]
Chronic heart failure	miR‐133a	Akt signaling pathway	miR‐133a mimics	Inhibits Akt signaling pathway	Improves cardiac function and reduces fibrosis	[213]
Atherosclerosis	miR‐22‐3p	HMGB2	SNHG16 knockdown	ceRNA mechanism promotes proliferation and migration	Inhibits plaque formation and progression	[214]
Atherosclerosis	miR‐320b	ABCG1/EEPD1 pathway	AAV‐mediated overexpression	Inhibits cholesterol efflux and promotes inflammation	Potential therapeutic target	[187]
Atherosclerosis	miR‐127‐3p	SCD1/UFAs pathway	miR‐127‐3p agonist/antagonist	Downregulates SCD1 to enhance proliferation and polarization	Potential therapeutic target	[188]
Atherosclerosis	miR‐576	KLF5	miR‐576 overexpression	Targets KLF5 to inhibit inflammation	Ameliorates malignant progression of atherosclerosis	[215]
Kawasaki disease	miR‐223	PDGFRβ	Platelet transfer/mimics	Inhibits PDGFRβ to promote differentiation	Enhances vascular repair and facilitates risk identification	[216]
Atrial fibrillation	miR‐1202	nNOS/TGF‐β1/Smad pathway	miR‐1202 inhibitor	Targets nNOS to promote fibrosis	Potential therapeutic target	[217]

Abbreviations: AAV, adeno‐associated virus; ADSC, adipose‐derived stem cell; ceRNA, competing endogenous RNA; CRISPR, clustered regularly interspaced short palindromic repeats; dCas9, dead CRISPR‐associated protein 9; eNOS, endothelial nitric oxide synthase; HAT, histone acetyltransferase; HDAC, histone deacetylase; HFpEF, heart failure with preserved ejection fraction; LDL, low‐density lipoprotein; miRNA, microRNA; MSC, mesenchymal stem cell; NAFLD, nonalcoholic fatty liver disease; nNOS, neuronal nitric oxide synthase; PDGFRβ, platelet‐derived growth factor receptor beta; RAS, renin–angiotensin system; SREBP, sterol regulatory element‐binding protein; TGF‐β1, transforming growth factor‐beta 1; UFAs, unsaturated fatty acids; VLDL, very low‐density lipoprotein.

### 6.3 Opportunities and Challenges: Target Specificity and Biodistribution

Therapies based on miRNA hold great promise for treating many human diseases. Despite this, their development into clinical application is hampered by two issues: limited target selectivity and uneven biodistribution. Using multiple targets, the miRNAs can modulate complex pathological signaling pathways. The ability to regulate gene expression and the subsequent therapeutic effects that it leads to is very beneficial. However, it can also lead to concerns of off‐target effects as well as nonspecific expression in various tissues. These can endanger the safety and efficacy of these strategies. A single miRNA can effectively target hundreds of other genes simultaneously. Furthermore, the function of miRNAs is context dependent. This means that the effects of miRNAs vary depending on tissue type, epigenetic changes, and pathological microenvironment. To circumvent off‐target interactions, LNA‐modified ASOs and miRNA mimics have been developed to increase sequence specificity and enhance molecular recognition.

**FIGURE 7:**
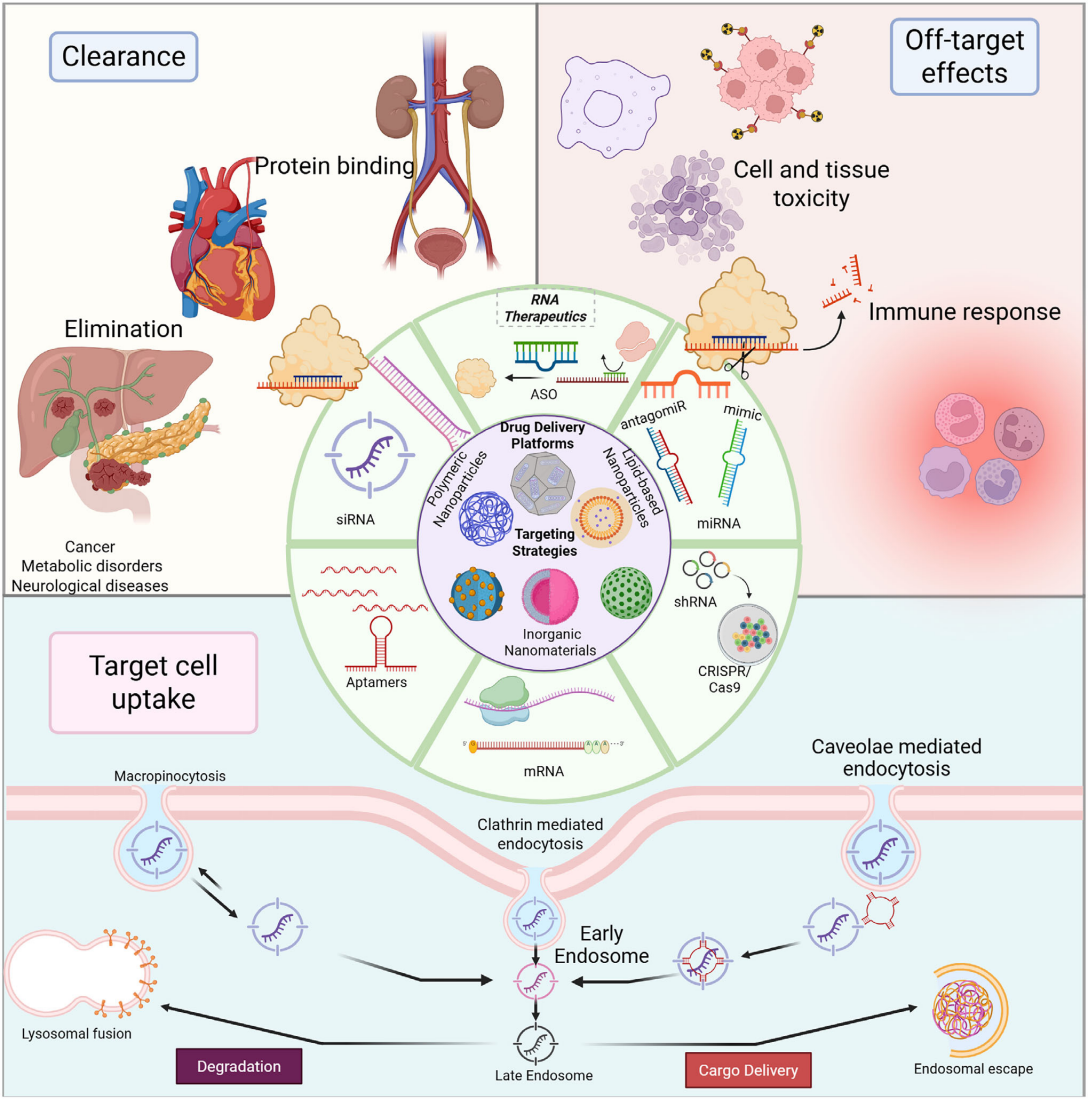
Systemic barriers, cellular uptake routes, and intracellular trafficking pathways influencing RNA therapeutic delivery. The figure outlines the principal challenges faced by RNA therapeutics, including their binding to plasma proteins, their clearance in the liver and spleen, their activation of the immune system, and their accumulation in unintended tissues. The study shows different pathways that include clathrin‐mediated, caveolae‐dependent, and macropinocytic uptake, which are involved in endosomal progression. Examples of RNA formats and delivery mechanisms are illustrated, showing how targeting approaches affect intracellular trafficking and safety issues. RNA, ribonucleic acid.

Another difficulty in clinical implementation is the suboptimal biodistribution and delivery efficiency of miRNA therapeutics. Free miRNAs are prone to degradation by nucleases. They can be quickly inactivated through plasma protein binding or rapid clearance by the liver and spleen. A comprehensive overview of these systemic barriers and cellular processes is presented in Figure 7. Hence, they possess low bioavailability with short systemic half‐lives. To overcome these challenges, an array of delivery platforms—LNPs, polymeric nanocarriers, or exosome‐based systems—have been designed for enhanced stability and tissue‐specific delivery. These approaches are not equally effective in all tissues. For example, LNPs preferentially aggregate in the liver, leading to off‐target accumulation, while transfer efficiency to organs such as the heart, brain, and skeletal muscle remains inadequate. To improve delivery specificity, investigators have designed targeting‐modified systems. Exosomes derived from dual‐modified mesenchymal stem cells and conjugated with TAT and RVG peptides deliver miR‐15b‐5p inhibitors effectively, reducing the infarct size and improving neurological deficits. The effectiveness of extracellular vesicles (EVs) was studied in an in vivo rat model of ischemia. The therapeutic effect was mediated by the exosomes modulating the HTR2C–ERK signaling pathway [218]. Subsequent confirmation with PET–MRI imaging sought to assess the ability of miRNA‐based therapeutics to traceably accumulate in metastatically involved sites including lymph nodes, lungs, and bone [219]. Lactobionic acid‐modified LNP formulations have also been shown to be effective in delivering camptothecin and miR‐145 simultaneously to hepatocellular carcinoma cells; improving drug retention and therapeutic efficacy [220].

Microenvironments related to diseases as well as tissue barrier integrity significantly influence the effectiveness of miRNA delivery. An example of this phenomenon is Coxsackievirus A16, which crosses the BBB and injures the CNS selectively by downregulating miR‐1303, which activates MMP9 [221]. Conversely, miR‐30c upregulation could inhibit glioma cell proliferation and migration [222]. On the other hand, an increase in miR‐142‐3p is believed to activate the Wnt pathway as well as mediate an increase in miR‐150 that leads to increased proliferation of tumor cells in breast cancer [223]. The expression of PTEN in osteoclasts is inhibited by miR‐19, an exosomal miRNA originating from tumors [224]. The results, taken together, indicate a dual functioning of miRNAs, which may show therapeutic or deleterious effects depending on their tissue localization and microenvironment context.

A key objective in miRNA therapy is to achieve a balance between specific targeting and controlled biodistribution. Future work will be done on optimal circulation time, optimal ligand‐to‐miRNA stoichiometric ratios, efficient cellular uptake, and codelivery of multiple miRNAs for synergistic pathway modulation [225]. Emerging chemical modification approaches such as phosphorothioate and 2′‐OME substitutions have generated highly stable miRNA mimics that exhibit prolonged in vivo half‐life and improved tissue specificity [226].

The main challenge for miRNA‐targeted therapy is achieving high target specificity and favorable biodistribution simultaneously. Optimized sequence engineering, improved delivery vehicle design, and the merge of near‐real‐time monitoring with intelligent regulatory systems allow miRNA therapeutics to potentially overcome existing technical bottlenecks positioned for translational development leading to precision clinical interventions for complex, multigenic diseases.

The translation of miRNA therapies will shift from the “single‐target” concept to a system‐level network regulation approach. Future strategies will integrate multiomics data with AI modeling to predict the complex downstream effects of miRNA modulation on cellular pathways. Advancements in exosome engineering and cell‐specific ligand conjugation will address bio‐distribution challenges, enabling precise delivery of miRNA mimics or inhibitors to damaged tissues. Ultimately, achieving spatiotemporal control of miRNA activity will be key to unlocking its potential in regenerative medicine and complex metabolic diseases.

## 7 shRNA and sgRNA‐Based Targeted Therapies

shRNAs and sgRNAs underpin two complementary RNA‐guided platforms—RNAi‐based knockdown and CRISPR/Cas‐based genome editing—for durable control of gene expression. This section first introduces the fundamental mechanisms of shRNA and CRISPR/Cas9 technologies, then summarizes disease‐targeted applications across metabolic, neurological, oncologic, and infectious diseases, and finally discusses key issues regarding editing efficiency, off‐target effects, and long‐term safety.

### 7.1 Fundamental Mechanisms of shRNA and CRISPR Technologies

The system of shRNA and sgRNA runs through two corner stone technologies related to RNA‐targeted therapeutics. These shRNA and sgRNA run through RNAi and genome editing. Both techniques require RNA hybridization to the specific sequences in their genetic target and can be used to control gene expression. These methods have proven useful to overcome genetic disorders, tumors, and communicable diseases.

shRNA mimics the natural RNAi mechanism. An RNA molecule is defined by a hairpin loop structure and is transcribed by RNA polymerase II or III. The processed transcript is processed by Drosha and Dicer to form siRNA. The mature siRNA that results from this process is incorporated into the RISC, which then degrades the mRNA or inhibits translation, causing gene silencing [227]. Unlike the transiently transfected siRNAs, vector‐based shRNA provides a more sustained gene knockdown and is ideal for chronic disease models and long‐term functional studies. The TET–ON system is one of the recent advances in inducible shRNA. By inserting a tetracycline‐responsive element into the promoter region, shRNA expression can be modulated by an external agent (tetracycline) for dynamic and reversible gene silencing. This invention greatly improves experiment controllability and allows spatiotemporal control of gene expression [228].

Unlike the previous case, here the CRISPR/Cas9 system acts on the genome to permanently modify it. The system consists of the Cas9 nuclease along with an sgRNA and evolved from immune defense strategies of archaea and bacteria. The sgRNA is a synthetic duplex of CRISPR RNA (crRNA) and trans‐activating crRNA that enables Cas9 to find sites along the genome with complementary sequences and a PAM sequence. The sgRNA guides Cas9 to cleave double‐stranded DNA by the RuvC and HNH nuclease domains, which causes double‐strand breaks. The nonhomologous end joining or homology‐directed repair (HDR) pathways repair these breaks to achieve gene knockout, knock‐in or precise correction of target loci. The CRISPR/Cas9 system quickly became the foremost gene‐editing platform due to its ease of use, customizability, and efficacy.

Both shRNA and CRISPR systems are valuable tools in functional genomics and phenotypic screening. Gene knockdown based on shRNA is a useful technique for broad‐spectrum genetic screens. For instance, they have been effective for identifying genes that modulate drug responses or cytotoxicity. One such example is key regulators of GSK983 sensitivity [229]. In contrast, complete knockout of nonessential genes with CRISPR/Cas9 technology is effective for nonessential gene function studies and disease pathways.

**FIGURE 8:**
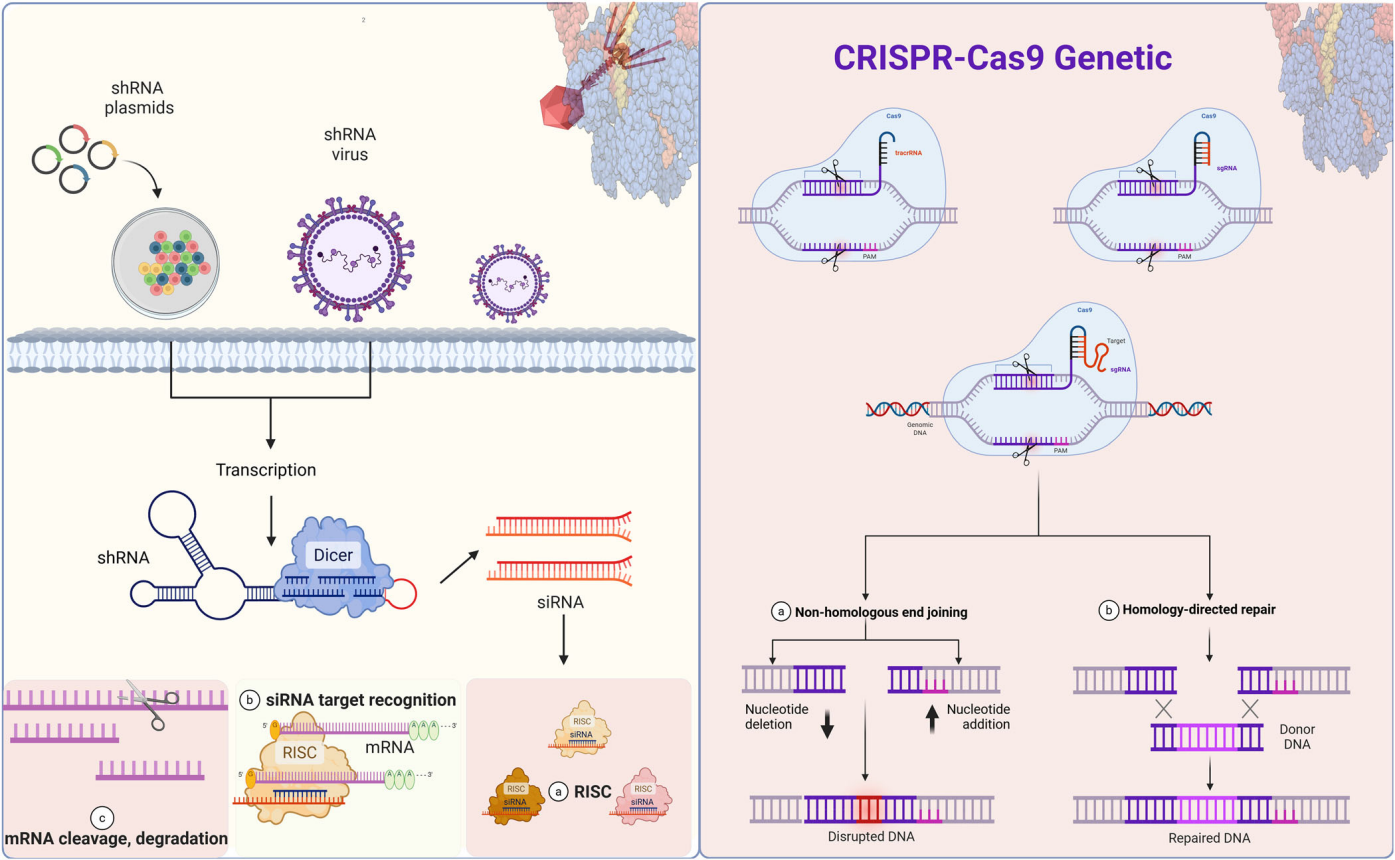
RNA‐guided gene silencing by shRNA and genome editing by CRISPR–Cas9. The left panel demonstrates how shRNA‐mediated RNAi works. Here, Dicer processes vector‐expressed shRNAs to generate siRNA duplexes. These are then loaded into the AGO‐containing RISC, leading to sequence‐specific cleavage of target mRNAs. The right panel shows CRISPR–Cas9‐mediated genome editing. Here, sgRNAs direct Cas9 specifically to complementary genomic sites to create DSBs. The DSBs are repaired through either nonhomologous end joining or HDR. shRNA, short hairpin RNA; RNAi, RNA interference; siRNA, small interfering RNA; AGO, argonaute; RISC, RNA‐induced silencing complex; mRNA, messenger RNA; sgRNA, single‐guide RNA; Cas9, CRISPR‐associated protein 9; DSB, double‐strand break; HDR, homology‐directed repair; CRISPR, clustered regularly interspaced short palindromic repeats.

The combination of shRNA and CRISPR technologies offers a broad range of molecular control, providing options to do reversible knock‐down or stable knock‐out. shRNA acts after transcription to regulate the expression of genes and can be a useful tool for dealing with gain‐of‐function mutations or genes that are pathologically overexpressed. In contrast, CRISPR/Cas9 acts directly on the genomic DNA and provides the opportunity to correct mutations, insert genes, and effect structural repairs. Recent studies also emphasize that chemical modification, delivery system design, and safety control are advancing rapidly to enable clinical translation of these approaches. They lay the groundwork for the rigorous, molecular treatment of complex diseases at their genetic source. As such, both shRNA and sgRNA play a significant role in the RNA therapeutic landscape. While shRNA permits reversible gene silencing, CRISPR/Cas9 provides permanent changes in the genomic makeup. Consequently, the use of both biological entities makes gene therapy versatile. Therefore, Figure 8 highlights the mechanistic pathways and molecular regulatory processes of shRNA and CRISPR/Cas9 in modeling diseases.

### 7.2 Disease‐Targeted Applications

The shRNA and CRISPR/Cas9 systems have progressed from basic experiments to preclinical translation studies, showing promise for gene silencing and mutation correction. The RNAi strategy through siRNA or shRNA enables continuous posttranscriptional gene silencing of aberrantly expressed genes. On the other hand, the CRISPR/Cas9 system can give rise to genomic cleavage and repair that fundamentally corrects pathogenic mutations. Together, these two strategies are complementary therapeutic platforms for genetic, metabolic, and oncogenic diseases.

In the case of metabolic and neurological disorders, RNA‐based therapeutic applications have shown tissue‐targeting and long‐term benefits. Chemically modified siRNA–GalNAc conjugates could provide potent hepatocyte‐specific delivery via receptor‐mediated endocytosis. They display strong hepatotropism and sustained therapeutic effects. They present a promising RNA drug platform for liver‐related diseases [230]. Through an intravitreal injection of a lipid‐modified siRNA, transthyretin expression can be inhibited in the retina. This delay in retinal degeneration has ocular applications [231]. In addition, self‐assembled EVs carrying LRRK2 siRNA can lower LRRK2 protein levels and improve neuropathological features in models of PD, demonstrating the potential of RNA therapeutics for neurodegenerative diseases [89]. Using siRNA with cerium oxide nanozymes also lessens PD pathology by downregulating reactive oxygen species and preventing aSyn aggregation. Thus, RNA therapy can regulate neuro‐metabolic dysfunction in a combined manner [232].

In cancer, shRNA and siRNA can effectively inhibit tumors through the silencing of multiple targets. A replicating oncolytic adenovirus carrying SATB1 shRNA inhibits the proliferation of prostate tumors and enhances their clearance by the immune system. These findings suggest a synergistic effect between RNAi‐based therapy and oncolytic virotherapy [233]. In a similar manner, nanoparticles loaded with anti‐RAN shRNA successfully transport RNA materials into breast cancer cells, effectively reducing the proliferation and migration of tumor cells [234]. Glioblastoma coadministration of B7H6‐siRNA with temozolomide augments chemosensitivity and therapeutic response, substantiating the promise of RNA‐based combination therapy [235]. In addition to cancer treatment, RNA delivery systems have shown promising results in tissue repair. For example, siMMP2‐loaded hydrogels significantly reduced extracellular matrix degradation and improved myocardial thickness in experiments with myocardial infarction [236].

Intervention through RNAi or CRISPR/Cas9 has been used effectively against several viruses. The replication of bovine herpesvirus 1 (BHV1) was significantly inhibited after lentivirus‐mediated shRNA targeting of the BHV1–UL25 was employed. This strategy demonstrates the principle. Similar strategies against HIV, HBV, and SARS‐CoV‐2 confirm the complementary potential of RNAi and CRISPR systems for antiviral therapy [237].

Overall, RNAi‐based systems (e.g., shRNA/siRNA) are ideal for treating conditions involving gene overexpression due to their stable, controllable, and long‐term silencing effect. In contrast, the CRISPR/Cas9 system, which enables permanent genome editing for mutation correction and functional restoration, is particularly suited to inherited disorders and immune‐related diseases. Owing to the continuous progress in RNA chemical modification, nanocarrier delivery, and off‐target effect control, the two mentioned RNA‐targeted strategies are driving clinical translation of precision molecular interventions while supplying new conceptual and technological foundations for personalized treatment of complex diseases. shRNA‐mediated RNAi and CRISPR/Cas9 (utilizing sgRNA) represent two major paradigms of RNA‐mediated genetic intervention, ranging from reversible gene silencing to permanent genome editing. Table 4 summarizes selected applications of shRNA and CRISPR/Cas9 systems, including representative target genes, disease models, mechanisms, and development status.

**TABLE 4:** Targeted therapeutic applications of shRNA and sgRNA in various diseases.

Disease type	Target RNA type	Target gene/pathway	Editing/interference method	Therapeutic mode and major effect	References
Acute myeloid leukemia	shRNA	SENP1	RNAi	Inhibits proliferation and induces apoptosis	[238]
Non‐small cell lung cancer	shRNA	YME1L	RNAi	Suppresses tumor growth and migration	[239]
Lung cancer	shRNA	FBXO9	RNAi	Inhibits migration and metastasis	[240]
Ovarian cancer	sgRNA	BARD1	CRISPR epigenetic editing	Enhances antiangiogenic therapy response	[241]
Non‐small cell lung cancer	shRNA	ORC6	RNAi	Suppresses tumor growth and migration	[242]
Glioma	shRNA	ORC6	RNAi	Inhibits tumor proliferation and growth	[243]
Nasopharyngeal carcinoma	shRNA	Gαi1	RNAi	Inhibits tumor proliferation and growth	[244]
Colorectal cancer	shRNA	AMPKα1/PKCα	RNAi	Suppresses tumor proliferation and growth	[245]
Breast cancer with lung metastasis	shRNA	TEAD4–FN1 axis	RNAi	Inhibits migration and lung metastasis	[246]
Non‐small cell lung cancer	shRNA	NDUFS8	RNAi	Inhibits tumor proliferation and growth	[247]
Castration‐resistant prostate cancer	shRNA	MTCH2	RNAi	Suppresses tumor growth and proliferation	[248]
Cutaneous T‐cell lymphoma	shRNA	JAK3–INSL3 fusion gene	RNAi	Inhibits tumor proliferation and growth	[249]
Glioma	shRNA	Gαi2	RNAi	Inhibits tumor proliferation and growth	[250]
α‐Herpesvirus infection	siRNA	USP14	RNAi	Suppresses viral replication	[251]
SARS‐CoV‐2 infection	shRNA	ORF7a	RNAi	Inhibits viral replication	[252]
HIV‐1 infection	sgRNA	SLTM	CRISPRi	Reactivates and eliminates latent virus	[253]
Ischemic stroke	shRNA	PTRF/PLA2G4A axis	RNAi	Reduces brain injury and neurological deficits	[254]
Neurological disorders	sgRNA	Ai9 reporter gene	CRISPR editing	Achieves efficient brain‐targeted editing	[255]
Impaired skin wound healing	shRNA	CCDC25 / ILK	RNAi	Inhibits fibroblast overactivation	[256]
Radiation‐induced pulmonary fibrosis	shRNA	DNA–PKcs/Twist1 pathway	RNAi	Inhibits epithelial–mesenchymal transition	[257]
Pathological angiogenesis	shRNA	TIMM44	RNAi	Suppresses retinal neovascularization	[258]
Hepatic encephalopathy	shRNA	EGLN3	RNAi	Reduces ammonia‐induced apoptosis	[259]
SARS‐CoV‐2 infection	siRNA	ORF1	RNAi	Inhibits viral replication and spread	[260]
Neuroblastoma	shRNA	EED	RNAi	Inhibits cell proliferation and colony formation	[261]
HCC	shRNA	Gαi2	RNAi	Suppresses tumor growth and proliferation	[262]
Breast cancer	shRNA	F9	RNAi	Regulates senescence and cell cycle arrest	[263]
Triple‐negative breast cancer	shRNA	REG3A	RNAi	Inhibits tumor growth and proliferation	[264]
Gastric cancer	siRNA	PDIA3	RNAi	Suppresses tumor proliferation and growth	[265]
EGFR‐overexpressing cancers	shRNA	DUSP1	RNAi	Enhances EGF‐induced apoptosis	[266]
Neovascular age‐related macular degeneration	shRNA	Wnt5a/β‐catenin pathway	RNAi	Inhibits subretinal fibrosis	[267]

Abbreviations: CRISPR, clustered regularly interspaced short palindromic repeats; CRISPRi, CRISPR interference; EGF, epidermal growth factor; EGFR, epidermal growth factor receptor; HCC, hepatocellular carcinoma; HIV, human immunodeficiency virus; RNAi, RNA interference; SARS‐CoV‐2, severe acute respiratory syndrome coronavirus 2; sgRNA, single‐guide RNA; shRNA, short hairpin RNA; siRNA, small interfering RNA.

### 7.3 Opportunities and Challenges: Efficiency of Editing and Unintended Effects

The development of shRNA and CRISPR/Cas9 technologies has opened new doors in the field of RNA‐targeted therapies. However, the major barriers to their clinical application remain editing efficacy and off‐target effects. Whether in RNAi‐based reversible gene silencing or CRISPR/Cas9‐mediated permanent genome editing, therapeutic success depends on the balance between target sequence specificity and intracellular editing efficacy. Improving therapeutic activity and decreasing nonspecific interactions remains a major scientific challenge for the safe and regulated use of RNA‐based therapeutics.

Various factors influence the performance of shRNA‐based regulation, including transcriptional activity and Dicer processing efficiency. Subsequently, this happens after the loading of the RISC complex. The structure's stability and shRNA hairpin configuration play a vital role in determining siRNA maturation rate and target mRNA degradation. The latest advancement, including algorithm optimization and high‐efficiency promoters (U6 and H1), has vastly improved transcriptional output and silencing potency. Furthermore, strategies that modulate signaling pathways can enhance therapeutic synergy. Despite this, the shRNA seed sequence can complement off‐target mRNAs that can lead to off‐target silencing, toxicity and metabolic perturbations. By using a low‐expression promoter system and bioinformatic prediction models the risk associated with shRNA can be effectively reduced.

The CRISPR/Cas9 system is flexible and able to create permanent genetic repairs, but it has serious issues with off‐target effects. The PAM sequence enables the sgRNA‐guided Cas9 complex to identify the target DNA sequence. However, many sequences in the human genome exhibit homology or partial matches to the sgRNA, which may lead to off‐target insertions, deletions, or mutations [268]. Thus, we must rationally design sgRNA for maximal editing specificity. Algorithms and computer tools can optimize sgRNA selection to improve target affinity while selecting against off‐target potential [269]. The EpiCas‐DL model, which integrates sequence with epigenetic data, predicts sgRNA activity in contexts of gene activation and repression, as an example [270]. Simultaneously, machine learning‐driven tools such as PAVOOC provide an in‐depth evaluation of on‐target and off‐target effects to identify sgRNA candidates with high specificity [271]. The inherent tolerance of Cas9 for mismatches or secondary structures can also lead to unintended DNA cleavage, chromosomal rearrangements, insertional mutations, or genomic instability [272]. Beyond molecular off‐target effects, CRISPR/Cas activity may trigger adaptive cellular responses with potential for cross‐tissue editing effects, posing additional biosafety challenges [273].

### 7.4 Integration Prospects of RNA Targeted Therapies and Gene Editing Technologies

With the rapid advancement of ADAR‐mediated RNA base editing, RNA‐targeted therapies are evolving from single regulatory methods to complex therapeutic systems centered on PTM interference (PTMi). The utility of RNA editing has been significantly enhanced with the improved SNAP‐ADAR tool. Its transient nature is a unique advantage, enabling rapid, dose‐dependent, and reversible modulation of gene expression at the RNA level. This contrasts sharply with DNA‐editing strategies like CRISPR/Cas, which make permanent changes to the genome. The reversibility of RNA editing provides a key advantage and natural complement to permanent genome‐editing strategies, safely targeting fundamental biological processes where permanent changes could lead to adverse effects, thus offering unique complementary potential in disease intervention [274].

Driven by advances in delivery technologies, the mRNA‐based Cas9/sgRNA system, with its transient expression characteristics, avoids the integration risks associated with viral vectors and reduces off‐target concerns. When combined with tissue‐specific LNP delivery, it can further confine editing activity to target organs, enhancing overall safety [275]. Additionally, RNA‐targeting tools can serve as “auxiliary regulators” within the editing window, enhancing editing efficiency and safety by reducing competing endogenous RNA, improving target exposure, or providing functional compensation when editing is incomplete. In contrast, RNA editing platforms do not require DNA cleavage, enabling precise nucleotide conversion. When combined with ASO‐like design strategies, they form a more controllable “reversible editing” framework, better suited for high‐safety scenarios.

Several studies suggest that combining LNP–mRNA editing systems with ASO/siRNA regulatory strategies can enable multilayered precision interventions in metabolic diseases, cancer, and genetic disorders. This cross‐platform integration is reshaping the treatment landscape of RNA‐based medicine, offering more adaptable molecular tool combinations for future personalized therapies.

Gene editing is advancing from simple disruption to precise rewriting. Technologies like Prime Editing and Base Editing are overcoming the limitations of double‐strand breaks, providing a safer therapeutic window. Simultaneously, AI‐driven off‐target prediction tools are becoming indispensable for verifying the clinical‐grade safety of sgRNAs. Future developments will focus on nonviral delivery vectors capable of carrying large editing machinery to specific somatic cells, ensuring that permanent genomic corrections are both efficient and restricted to target tissues.

## 8 Preclinical and Clinical Development of RNA Targeted Therapies

Increasing preclinical evidence has established the translational potential of RNA‐targeted therapies in neurological, cardiovascular, metabolic, infectious, and oncological diseases. In rodent and large animal models, ASOs and siRNAs have been shown to dose‐dependently suppress pathogenic transcripts, rescue synaptic and motor function, and extend survival in single‐gene neurodegenerative disease models. In the SOD1 mutant ALS model, novel bivalent siRNAs demonstrated survival benefits superior to existing ASO strategies [276]. Chemically modified miRNA mimics and inhibitors have been used to regulate complex signaling networks in cancer and inflammation, while aptamers and shRNA vectors have achieved sustained pathway modulation and targeted delivery in tumors and models [277, 278]. Additionally, the mRNA–LNP platform has shown powerful in vivo protein expression, antigen presentation, and immune activation capabilities in infectious disease and cancer vaccine models. Overall, these animal studies bridge mechanisms between RNA biology, target binding, and disease modification, guiding dose selection, safety monitoring, and biomarker strategies for subsequent human trials.

Building on this, various RNA‐based therapies have entered late‐stage clinical development and received regulatory approval. The GalNAc‐conjugated ASO drug targeting APOC3, Olezarsen, reduced triglyceride levels and decreased acute pancreatitis events in patients with familial chylomicronemia syndrome in the phase 3 BALANCE trial (NCT04568434). It is expected to become the first approved RNA therapy for this severe metabolic disorder [279]. The siRNA drug zerlasiran, in Phase 2 trials (NCT05537571), significantly reduced lipoprotein(a) levels in patients with stable ASCVD by targeting hepatic synthesis of apolipoprotein(a). After multiple doses, it showed a significant reduction in the time‐averaged concentration of lipoprotein(a) compared with the placebo group, with good tolerability and the most common adverse event being injection site reactions. This study suggests that zerlasiran, as a targeted therapy, could provide a new approach for reducing cardiovascular disease risks associated with lipoprotein(a) [280]. mRNA therapies have achieved a milestone in cancer immunotherapy: the personalized neoantigen mRNA cancer vaccine mRNA‐4157 (V940), in combination with pembrolizumab, significantly reduced the risk of recurrence or death by approximately 43.9% in high‐risk melanoma patients in the phase 2b KEYNOTE‐942 trial (NCT03897881), confirming the immense potential of mRNA–LNP systems in developing precision cancer immunotherapies [281]. To systematically summarize the latest progress in the clinical application of nucleic acid therapies (including RNA drugs and gene therapies), we have compiled key drugs that are currently approved or in Phase III clinical trials. Table 5 details the category (e.g., siRNA, ASO, mRNA vaccines, etc.), precise mechanism of action, indication(s), and the latest clinical development status of these drugs, providing a comprehensive and authoritative overview of clinical advances.

**TABLE 5:** Summary table of clinical progress in nucleic acid therapies (RNA drugs and gene therapies).

Drug name	RNA class/type	Mechanism of action	Indication	Clinical development status	References
Patisiran	siRNA	Inhibits hepatic synthesis of transthyretin, reducing the production of pathogenic transthyretin protein	Hereditary transthyretin amyloidosis with polyneuropathy; ATTR cardiomyopathy	Approved; Phase III trial shows improvement in multiple clinical manifestations	[282, 283]
Inclisiran	siRNA	Inhibits hepatic synthesis of PCSK9 protein, leading to increased LDL receptor expression and lower LDL‐C	Hypercholesterolemia (HeFH, ASCVD); patients with inadequate response to maximally tolerated statin therapy	Approved; Phase III trial shows significant and sustained lipid lowering	[284, 285]
mRNA‐1273	mRNA	Encodes the full‐length spike protein of SARS‐CoV‐2	Prevention of COVID‐19	Phase III trial shows 94.1% efficacy	[8]
Nusinersen	ASO	Modulates SMN2 pre‐mRNA splicing to promote expression of full‐length, functional SMN protein	SMA	Approved; Phase III CHERISH trial shows significant improvement in motor function	[286, 287]
Onasemnogene abeparvovec	Gene therapy (AAV9)	Delivers SMN cDNA via AAV9 vector to enable motor neurons to express SMN protein	SMA	Approved; Phase III trial shows near‐normal motor development	[288, 289]
Givosiran	siRNA	Inhibits hepatic ALAS1 gene expression, reducing neurotoxic porphyrin precursors	AHP, including acute intermittent porphyria	Approved; long‐term treatment significantly reduces annualized attack rate	[290, 291, 292, 293]
Lumasiran	siRNA	Targets glycolate oxidase to reduce hepatic oxalate production	PH1	Approved; Phase III and long‐term studies show efficacy and safety	[294, 295, 296]
Volanesorsen	ASO	Inhibits ApoC‐III synthesis to lower plasma triglycerides	Mixed hypertriglyceridemia	Phase III trial	[297]
Fitusiran	siRNA	Targeted inhibition of antithrombin to rebalance the hemostatic system	Hemophilia A or B with or without inhibitors	Phase III trial	[298]

Abbreviations: AAV, adeno‐associated virus; AHP, acute hepatic porphyria; ASCVD, atherosclerotic cardiovascular disease; ASO, antisense oligonucleotide; ATTR, transthyretin amyloidosis; cDNA, complementary DNA; HeFH, heterozygous familial hypercholesterolemia; LDL, low‐density lipoprotein; LDL‐C, low‐density lipoprotein cholesterol; mRNA, messenger RNA; PCSK9, proprotein convertase subtilisin/kexin Type 9; PH1, primary hyperoxaluria Type 1; SARS‐CoV‐2, severe acute respiratory syndrome coronavirus 2; siRNA, small interfering RNA; SMA, spinal muscular atrophy; SMN, survival motor neuron.

## 9 Discussion

The rapid evolution of RNA‐targeted therapies is reshaping the intervention paradigm in molecular biology, transitioning from traditional protein function‐based regulation models to a novel framework grounded in gene expression and RNA dynamic networks. These nucleic acid drugs span a complete action axis, from sequence recognition (ASO/siRNA) to directed expression (mRNA/circRNA) and genomic editing (CRISPR), enabling multilayered precise interventions at the transcriptional, translational, and genomic levels. However, key challenges remain in enhancing delivery efficiency, improving in vivo stability, and ensuring biosafety, which continue to impede the clinical translation of RNA‐based therapies [299].

Molecular stability and membrane penetration efficiency are the key limiting factors for the efficacy of all RNA drug formats. Endogenous RNA is unstable due to degradation by intracellular nucleases and repulsive interactions with cellular membranes caused by its strong negative charge. This means these drugs have to use delivery systems like LNPs, GalNAc, polymer nanoparticles, and engineered exosomes to provide nucleic acids with protection, charge shielding, and intracellular delivery [300]. Although these carriers can enhance tissue entry efficiency, they introduce new constraints themselves, such as accumulation in the liver, immune activation, or insufficient endosomal escape. Additionally, the structural properties of RNA, such as the presence of a 5′‐triphosphate or blunt‐ended duplex, can induce conformational changes in RIG‐I and facilitate CARD domain release, initiating interferon responses [301]. Due to this sensitivity to RNA structure and the likelihood of TLR and PKR activation, managing the immune response is an important step in RNA drug development that needs to be repeatedly addressed. In the last 10 years, the implementation of many new strategies such as 2′‐modification, LNA, immune‐evasive bases, and lipidation has greatly improved the stability and safety of these therapies. Nevertheless, there remains a fine balance between “enhancing activity” and “suppressing immune responses.”

From a technical perspective, different RNA platforms exhibit distinct functional characteristics. Table 6 summarizes and compares the characteristics of the current mainstream RNA‐targeted therapy platforms (including ASO, siRNA, mRNA, aptamer, and CRISPR/sgRNA). The table details the mechanism of action, key advantages, critical limitations, and representative application scenarios for each platform, aiming to provide readers with a clear reference for platform comparison and clinical potential. ASOs and siRNAs possess high sequence specificity but are heavily reliant on chemical modifications and delivery systems; siRNAs have a pronounced advantage in liver accumulation, yet delivery to the CNS and heart remains complex. The programmable format and capacity for rapid in vitro synthesis of mRNA give it an unusual position in the vaccine and protein therapy fields. Nevertheless, the presence of its own 2′‐hydroxyl group makes it chemically unstable and, due to the trigger of innate immunity, there is a risk. While mRNA is protected by LNPs, the LNPs themselves can degrade during production and storage, forming inactivated mRNA–LNP complexes. The combined factors mentioned above make developing mRNA formulations more difficult than developing formulations for oligonucleotide drugs, which are already stable in liquid form and under cold storage [302]. Aptamers have a high affinity and are less immunogenic than antibodies; however, their inherent instability under in vivo conditions limits their use. To tackle nucleases degradation and fast clearance, specific chemical modification (3′‐idT capping, etc.) or building a nanoparticle platform has become a key strategy to sustain the therapeutic activity [303]. With respect to stability, immune risk, tissue targeting, and production costs, these RNA platforms are not readily substitutable for one another, but they are clearly complementary, thus indicating prospects for enhanced cross‐platform combinatorial strategies. Furthermore, CRISPR/sgRNA platforms may offer long‐lasting gene correction; however, they present major technical challenges, including issues regarding delivery efficiency and potential off‐target effects.

**TABLE 6:** Comparative characteristics of RNA targeted therapy platforms.

Category (platform)	Mechanism of action	Primary/key advantages	Critical limitations	Representative application scenarios	References
ASO	Hybridizes with target RNA, inducing RNase H‐mediated degradation or modulating splicing	High sequence specificity; mature mechanism of action; can target specific mutation sites	Dependence on delivery systems; potential activation of certain immune receptors; requires repeated dosing	Genetic disorders, metabolic diseases, neurological disorders, and as an antitumor therapeutic	[14, 15, 16, 19, 20, 21, 22, 23, 36, 37, 38]
siRNA	RNAi–RISC mediated mRNA degradation	Prominent liver‐targeting advantage; high silencing efficiency; rich clinical experience	Difficulty in crossing the BBB/myocardial delivery; double‐stranded RNA may activate innate immunity	Liver diseases, metabolic disorders, antiviral applications, tumor suppression	[44, 45, 46, 47, 48, 49, 91, 92, 93, 94, 95, 96, 97]
mRNA	Translated into target protein in the cytoplasm	Programmable expression; short manufacturing cycle; strong platform versatility	Poor stability; high immunogenicity; strict reliance on cold chain for storage	COVID‐19 vaccine, protein replacement therapy, tissue repair	[118, 119, 120, 121, 122, 128, 129, 130, 168, 169, 170]
Aptamer	Specific recognition of proteins or receptors via three‐dimensional folded structure	High affinity; extremely low immunogenicity; easy in vitro screening	Short half‐life; susceptible to degradation; requires chemical modifications and carrier support	Targeted drug delivery, receptor blockade, anticoagulation, theranostics	[98, 99, 100, 104, 105, 106, 107, 108, 109, 110, 111]
CRISPR/sgRNA	sgRNA guides the Cas system for gene editing or transcriptional regulation	Enables long‐term gene correction; high precision/accuracy	Off‐target risk; significant delivery challenges; requires nuclear entry	Gene editing for genetic diseases, tumor therapy, long‐term gene modulation	[227, 228, 229, 239, 240, 241, 242, 268, 269, 270, 271, 272, 273]

Abbreviations: ASO, antisense oligonucleotide; BBB, blood–brain barrier; CRISPR, clustered regularly interspaced short palindromic repeats; mRNA, messenger RNA; RISC, RNA‐induced silencing complex; RNAi, RNA interference; RNase H, ribonuclease H; sgRNA, single‐guide RNA; siRNA, small interfering RNA.

RNA drug development is accelerating owing to multiomics analysis and AI as research progresses. According to omics data, RNA accessibility, tissue microenvironment, and immune recognition can help in predicting posttranscriptional regulatory effects and in vivo distribution patterns. Machine learning models find widespread application in designing ASOs/siRNAs, predicting off‐target effects, modeling sequence–structure relationships, and optimizing delivery systems. These models greatly enhance design accuracy and help liberate RNA drug development from the constraints of traditional trial‐and‐error, enabling more efficient and quantitative design [304]. The future of RNA research paradigms will be transformed by AI. AI is useful not only for improving the accuracy of efficacy predictions and shortening drug development times through RNA structure prediction and systems pharmacology modeling. For example, it is helpful in the interpretation of RNA regulatory logic at the whole transcriptome scale with the PlantRNA‐FM model. Most importantly, AI‐driven sequence design can now predict how modulating the movement rates of ribosomes and cotranslational folding pathways will enable the directed optimization of protein PTM patterns to maximally enhance the stability and functional activity of therapeutic proteins. The design goal for RNA drugs will shift from a single‐gene target strategy to a wider goal of remodeling the entire cellular system by regulating key nodes like translation dynamics [305]. Further, thanks to the programmability and network regulatory capabilities of RNA drugs, they have a natural advantage in the era of precision medicine. The authors also noted that miRNAs can also modulate multiple signaling networks at the same time, mRNA can modulate immune activation and tissue repair, and CRISPR/sgRNA can achieve long‐term gene correction. RNA therapies are rapidly evolving from “single‐target repairs” to “multipathway remodeling” and “microenvironment regulation.” These therapies are being studied extensively in cancer and complex, multisystem diseases after being used in single‐gene genetic diseases and infectious diseases [306]. The effective incorporation of these new modalities into cancer care will require adherence to biomarker‐driven, multidisciplinary management protocols; the standard‐of‐care algorithms for Stage III NSCLC exemplify this process [307]. The targeting of cancer stem cells is a powerful example showing how ncRNA‐mediated epigenetic networks can now be directly modulated by ASO, siRNA, and mRNA‐based epigenetic editors [308].

Therapeutics aimed at RNA are evolving from individual molecular instruments to a system‐wide therapeutic framework that can alter gene regulatory networks [309]. The combination of AI molecular design with next‐generation nanocarrier engineering will further improve precision, tissue specificity, and therapeutic durability. As advances converge, they will help RNA therapeutics to become a truly programmable and clinically relevant therapeutic modality.

## Author Contributions

Wangzheqi Zhang, Aimin Jiang, Bin‐Kui Jia, Yuming Jin, and Yinghu Chen contributed equally to this work and were involved in the conception and drafting of the manuscript. Zhaoyu Li and Yan Liao contributed to literature review and data collection. Haoling Zhang and Zhiheng Lin provided critical revision of the manuscript. Xiao Fang and Linhui Wang supervised the study and approved the final version of the manuscript. All authors have read and approved the final version of the manuscript.

## Funding

This research was funded by the National Natural Science Foundation of China (grant numbers: 81902560, 81730073), Shanghai Municipal Health Commission Science Research Project (20214Y0320) and the Naval Medical University Basic Medical Science Research Program (2022MS028).

## Ethics Statement

The authors have nothing to report.

## Conflicts of Interest

The authors declare no conflicts of interest.

## Data Availability

The authors have nothing to report.
